# The role of bone marrow microenvironment (BMM) cells in acute myeloid leukemia (AML) progression: immune checkpoints, metabolic checkpoints, and signaling pathways

**DOI:** 10.1186/s12964-023-01282-2

**Published:** 2023-09-21

**Authors:** Maryam Bakhtiyari, Mahsa Liaghat, Fatemeh Aziziyan, Hooriyeh Shapourian, Sheida Yahyazadeh, Maedeh Alipour, Shaghayegh Shahveh, Fahimeh Maleki-Sheikhabadi, Hossein Halimi, Razieh Forghaniesfidvajani, Hamidreza Zalpoor, Mohsen Nabi-Afjadi, Majid Pornour

**Affiliations:** 1 Department of Medical Laboratory Sciences, Faculty of Allied Medicine, Qazvin University of Medical Sciences, Qazvin, Iran; 2 Network of Immunity in Infection, Malignancy & Autoimmunity (NIIMA), Universal Scientific Education & Research Network (USERN), Tehran, Iran; 3 Department of Medical Laboratory Sciences, Faculty of Medical Sciences, Kazerun Branch, Islamic Azad University, Kazerun, Iran; 4 Department of Biochemistry, Faculty of Biological Sciences, Tarbiat Modares University, Tehran, Iran; 5 Department of Immunology, Faculty of Medicine, Isfahan University of Medical Sciences, Isfahan, Iran; 6 Department of Immunology, School of Medicine, Shiraz University of Medical Sciences, Shiraz, Iran; 7 Cellular and Molecular Biology Research Center, Health Research Institute, Babol University of Medical Sciences, Babol, Iran; 8 American Association of Naturopath Physician (AANP), Washington, DC USA; 9 Department of Hematology and Blood Banking, School of Paramedical Sciences, Shiraz University of Medical Sciences, Shiraz, Iran; 10 Shiraz Neuroscience Research Center, Shiraz University of Medical Sciences, Shiraz, Iran; 11 Department of Biochemistry and Molecular Biology, University of Maryland, Baltimore, MD USA; 12 Marlene and Stewart Greenebaum Comprehensive Cancer Center, Baltimore, Maryland, USA

**Keywords:** Acute myeloid leukemia, Bone marrow microenvironment, Cancer metabolism, Metabolic checkpoint, Immune checkpoint, Angiogenesis, Chemoresistance

## Abstract

Acute myeloid leukemia (AML) comprises a multifarious and heterogeneous array of illnesses characterized by the anomalous proliferation of myeloid cells in the bone marrow microenvironment (BMM). The BMM plays a pivotal role in promoting AML progression, angiogenesis, and metastasis. The immune checkpoints (ICs) and metabolic processes are the key players in this process. In this review, we delineate the metabolic and immune checkpoint characteristics of the AML BMM, with a focus on the roles of BMM cells e.g. tumor-associated macrophages, natural killer cells, dendritic cells, metabolic profiles and related signaling pathways. We also discuss the signaling pathways stimulated in AML cells by BMM factors that lead to AML progression. We then delve into the roles of immune checkpoints in AML angiogenesis, metastasis, and cell proliferation, including co-stimulatory and inhibitory ICs. Lastly, we discuss the potential therapeutic approaches and future directions for AML treatment, emphasizing the potential of targeting metabolic and immune checkpoints in AML BMM as prognostic and therapeutic targets. In conclusion, the modulation of these processes through the use of directed drugs opens up new promising avenues in combating AML. Thereby, a comprehensive elucidation of the significance of these AML BMM cells' metabolic and immune checkpoints and signaling pathways on leukemic cells can be undertaken in the future investigations. Additionally, these checkpoints and cells should be considered plausible multi-targeted therapies for AML in combination with other conventional treatments in AML.

Video Abstract

**Supplementary Information:**

The online version contains supplementary material available at 10.1186/s12964-023-01282-2.

## Introduction

Acute myeloid leukemia (AML) is a heterogeneous group of hematologic diseases characterized by the proliferation, blockade of differentiation, accumulation of leukemic cells in bone marrow (BM), and disturbance of normal hematopoiesis. The absence of treatment could lead to the rapid progression of AML which can be fatal in weeks to months [1–4]. AML is the most common acute leukemia and accounts for approximately 80% of cases in adults with 5-year survival rates below 20% for patients between 60 and 74 age [5]. Initial remission can be obtained in 30–40% of young patients after the standard induction chemotherapy regimens known as 7 + 3 (7 days infusion of Cytarabine and 3 days of Daunorubicin) [6]. Despite extensive attempts and breakthroughs in treatment during the last decades, the prognosis of AML particularly in older individuals remains the main challenge [7]. Therefore, recognition of alternative and novel therapies has become a research hotspot.

The important role of the bone marrow microenvironment (BMM) in the underpinning of normal hematopoiesis was first described by Schofield in 1998 [8]. BMM consists of cellular and molecular components whose interactions are essential to induce the fate of hematopoietic stem cells (HSCs) and has an important effect on the proliferation, self-renewal, and differentiation of these cells [9]. The cellular part comprises different cell types including stromal cells, endothelial cells, osteoblasts, adipocytes, Schwann cells, and immune cells, while the molecular components include cytokines, chemokines, growth factors, and matrix proteins. This affluent environment could also be profitable for malignant hematopoietic cells. The malignant BM creates a special microenvironment supporting the maintenance of cancer cells and tumor progression through cross-talk with tumor cells. The footprint of BMM in boosting leukemogenesis and survival of leukemia cells is provided in various research [10, 11].

A better understanding of events that occur in the BMM of leukemic cells and targeting these interactions could lead to a promising strategy for more efficient treatment, prolonging overall survival (OS) and increasing the life expectancy of AML patients. Therefore, in this review, we highlight the protective role of the BMM elements in the survival and progression of AML cells by focusing on immune checkpoints expression and production of BMM elements, signaling pathway mediation as well as metabolic adaptation of AML cells. Moreover, we discuss therapeutic prospective targets and prognostic insights to ameliorate the consequences of AML.

## Bone marrow microenvironment cells in AML: immune checkpoints (ICs) and component production

**Fig. 1:**
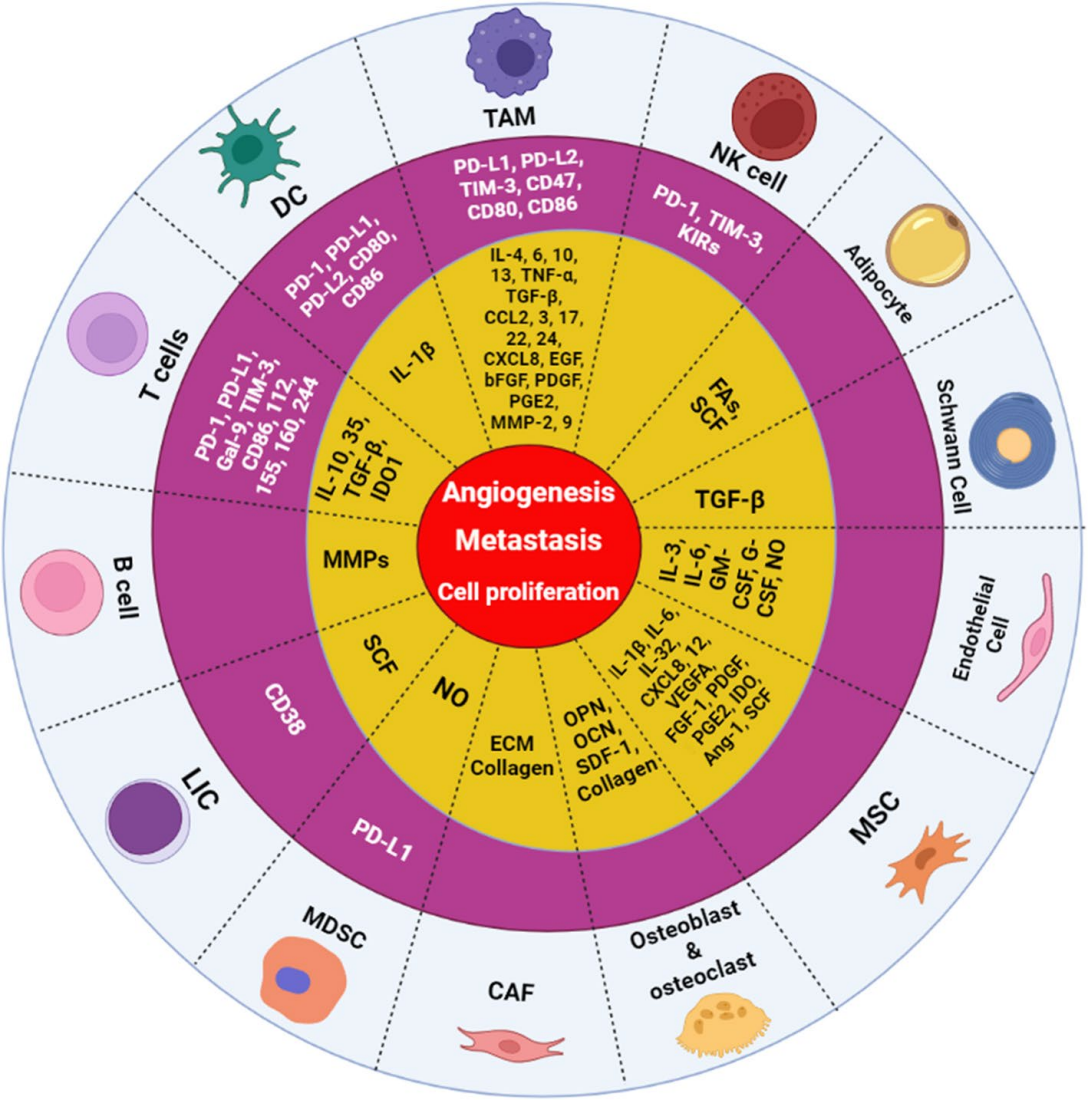
AML bone marrow microenvironment (BMM) cells’ immune checkpoints and component productions that contribute to angiogenesis, metastasis, and cell proliferation. The yellow concentric circle refers to cells component productions such as chemokines, cytokines, and growth factors. The purple concentric circle refers to immune checkpoints. TAM: tumor-associated macrophage; DC: dendritic cells; LIC: leukemia-initiating cell; MDSC: myeloid-derived suppressor cell; CAF: cancer-associated fibroblast; MSC: mesenchymal stromal cell; NK cell: natural killer cell; TNF-α: tumor necrosis factor-alpha; IL: interleukin; TGF-β: transforming growth factor-β; CCL: C–C motif chemokine ligand; CXCL: chemokine (C-X-C motif) ligand; NO: nitric oxide; SCF: stem cell factor; MMPs: matrix metalloproteinases; Ang: angiopoietin; IDO: indoleamine 2,3-dioxygenase; OPN: osteopontin; OCN: osteocalcin; VEGF: vascular endothelial growth factor; PDGF: platelet-derived growth factor; SDF: stromal cell derived factor; FGF: fibroblast growth factor; TIM-3: T-cell immunoglobulin and mucin domain-3; PD-1: Programmed Cell Death Protein 1; PD-L1: Programmed Cell Death Ligand 1; PGE2: Prostaglandin E2

Immune checkpoints (ICs) expressed by AML BMM cells have a significant role in AML progression. Furthermore, the BMM is composed of distinct components including soluble factors such as chemokines, cytokines, and growth factors which serve a variety of functions. These molecular components contribute to the proliferation and differentiation of hematopoietic stem/progenitor cells (HSPCs) and the maturation of cell lineages. A growing number of observations have proved the supporting role of BMM components in cancer progression. The relationship between cancer cells and components of the tumor microenvironment (TME) triggers cancer cell survival, angiogenesis, metastasis, proliferation, and cancer evading. Here we will focus on the different ICs and components which are expressed by various AML BMM cells, and their role in AML progression (Fig. 1). We suggest that the significance of these BMM cells on leukemic cells must be comprehensively elucidated in forthcoming investigations and regarded as plausible multi-targeted therapies.

### Tumor associated macrophage (TAM)

Several studies have highlighted the contribution of macrophages in forming a favorable leukemic microenvironment via multiple mechanisms including the production of various mediators. In the leukemic BMM, there are active interactions between tumor-associated macrophage (TAM) and malignant hematologic cells. As a result, these TAMs present an immunosuppressive and pro-tumorigenic phenotype, which can accelerate tumorigenesis [12]. The correlation between the higher number of TAMs and the poor prognosis of malignancies as well as AML has been shown by several studies [13]. Macrophages support the maintenance of progenitor cells in the bone marrow niche, which could be an important mechanism in the early stages of AML. Depending on the microenvironment in which macrophages were located, they displayed different phenotypes. Macrophages can be categorized into classical (M1) and alternative (M2) types of activated macrophages based on their involvement in inflammatory responses against pathogens and cancer cells and stimulation of wound healing and cancer progression, respectively. According to the polarizing cytokines of macrophages, M2 macrophages have been further sub-categorized to M2a, M2b, M2c, and M2d. In spite of a good comprehension of the M2 macrophages activities in solid tumors, the interactions between BM M2 macrophages and leukemic cells remain less understood [14]. Studies have revealed that BM from AML patients contained higher levels of M2 macrophages than controls and patients with high M2 accumulation appear to have a poorer prognosis [15, 16]. M2 macrophages have anti-inflammatory characteristics and can be stimulated by interleukin 4 (IL-4) or IL-13. They release matrix metalloproteinases (MMPs), arginase, transforming growth factor-β (TGF-β), IL-10, and other immune suppressor cytokines, which in turn, lead to angiogenesis, cancer progression, and tissue repair [15]. The primary role of TAMs in metastasis is associated with MMPs. MMPs include zinc-dependent endopeptidases with a critical role in tumor invasion. Studies have revealed that the MMP-9 and MMP-2 expression is related to leukemia progression [17, 18]. Aref S et al. have shown that a higher level of MMP-2 is correlated with shorter survival of AML patients [19]. Following this, TAMs activate signal transducer and activator of transcription (STAT) 3 in malignant cells and enhance the survival and proliferation of tumor cells.

M2 macrophages in solid tumors suppress anti-tumor immune responses and promote various malignant behaviors, including tumor invasion, angiogenesis, metastasis, and tumor recurrence [20]. However, further investigation is required for clarifying the specific pathological contribution of M2 macrophages to AML.

Co-stimulatory and inhibitory ICs/ligands are expressed by macrophages in AML include T-cell immunoglobulin and mucin domain-3 (TIM-3) [21], PD-L1, PD-L2 [22], and CD80, CD86, respectively [23]. Macrophages, in particular TAMs, express the CD47 immune checkpoint molecule, which has a noticeable role in the cancer cells’ immune escape mechanisms. As a signaling protein, CD47 was originally considered to be anti-phagocytic. CD47 impedes phagocytosis through attaching to its receptor, Signal Regulatory Protein α (SIRPα) on macrophages [24, 25]. Previous studies have shown that CD47 over-expression occurs in various tumors, like non-Hodgkin’s lymphoma, bladder cancer, breast cancer, as well as AML. In addition, clinical studies have shown promising outcomes for CD47-targeted therapies in AML as monotherapy and along with other treatment methods [23, 25].

A study demonstrated that CD206 as an M2 marker gene accurately reflected M2 fractional variation and was significantly expressed with high levels in AML patients compared to normal controls [15]. Additionally, CD200, as a poor prognostic factor, has been found to have an immunosuppressive impact on macrophages in AML [26].

TAMs can elevate tumor hypoxia and glycolysis, which are essential for angiogenesis [27]. Mortensen et al. have demonstrated that in AML rats model, as the disease progresses, the BMM changes, and one of these alterations is hypoxia [28]. Hypoxia-inducible factor-alpha (HIF-1α) is a protein that responds to hypoxia and regulates the expression of angiogenesis-growth factors like EGF, bFGF, PDGF, and prostaglandin E2 (PGE2) in TAMs. Several studies indicated that HIF-1α is overexpressed in AML cases and can be a prognostic indicator for patients. On the other hand, TAMs can support angiogenesis through the generation of pro-angiogenic factors, e.g., chemokine (C-X-C motif) ligand (CXCL8) and Semaphorin 4D (Sema4D) [29]. Secretion of CCL2 and CXCL8 from TAMs via the ER/Golgi secretory pathway and interaction with CCR2 and CXCR2, respectively, result in several downstream signaling events in the leukemic blasts and cause the activation of pro-survival pathways and inhibition of apoptosis [30]. The high expression of TAM-induced Sema4D is expected in a various types of cancers and is associated with tumor growth and invasion [31]. Hongchao Jiang et al. have reported that the overexpression of Sema4D in AML is related to poor prognosis [32].

Continuing the role of TAMs in the development of malignancy, it should be noted that TAMs lead to chemo-resistance in different ways. Through regenerative processes that are part of wound healing, TAMs contribute to tumor relapse. Accordingly, TAMs are responsible for secreting TNF-α, IL-6, Cathepsin B and S, and affect other cells to produce IL-6. Furthermore, suppression of immunity is also associated with chemo-resistance. The immunosuppressive activity of TAMs is due to the production of IL-10, and TGF-β resulting in the generation of induced Treg (iTreg) by the upregulation of the regulatory transcription factor forkhead box P3 (FOXP3) in CD4^+^ T cells [33]. Besides, TAMs can secrete CCL17, CCL22, and CCL24, which are related to Th2, and inhibition of inflammatory responses [34].

On the other hand, macrophages secrete a chemotactic chemokine called Macrophage inflammatory protein-1α (MIP-lα/CCL3). It has been found that there is an association between greater levels of MIP-1α and hematologic problems, like chronic lymphocytic leukemia (CLL), multiple myeloma (MM), as well as chronic myeloid leukemia (CML) [35]. Ping Lu et al. used MLL-AF9-prompted AML mouse model and showed that MIP-1a promotes the progression of AML [36].

Taken together, based on the crucial functions of TAMs in AML progression, angiogenesis, metastasis, and cell proliferation, it is imperative that the significance of each ICs and component productions on leukemic cells are comprehensively elucidated in forthcoming investigations, thereby considering them as plausible targeted therapies.

### Natural killer (NK) cells

Multiple mechanisms allow AML to escape from natural killer (NK) cell immunity, leading to the failure of the anti-leukemic immune response and one of them is NK cell abnormality [37]. It has been reported that AML-derived NK cells (AML-NK) express natural cytotoxicity-activating receptors (NCRs) weaker than NK cells derived from healthy donors [38]. NK cells express inhibitory ICs such as PD-1 [39], TIM-3 [40], and inhibitory killer cell immunoglobulin-like receptors (KIRs) [41]. The PD-1 by NK cell expression has been suggested as a result of trogocytosis [42].

KIR2DL4 receptors on NK cells interact with HLA-G, which is a non-classical HLA class I molecule, which controls immune response in pregnancy, transplantation, and autoimmune diseases. However, HLA-G plays a deleterious role in malignancies. It is thought that tumor cells are protected from NK cells and cytotoxic T lymphocytes by expressing HLA-G, which is a tumor-induced immune escape mechanism. Patients with relapsed AML had significantly higher levels of soluble HLA-G than controls [43]. Moreover, recent studies revealed that the increased mRNA levels of KIR2DL1, KIR2DL3, KIR2DL4, KIR3DL1, and KIR3DL2 are significantly associated with poor prognosis and overall survival (OS) for AML patients. In contrast, the KIR2DS4 mRNA levels did not have a prognostic significance [44]. Additionally, CD200 as a poor prognostic factor has been found to have an immunosuppressive impact on NK cells in AML [26]. RANK/RANKL axis in the bone can affect NK cells and it is substantial in metastasis formation. The cross-talk between NK cells and BMM through RANK/RANKL axis contributes to the release of NK-suppressing factors and leads to bone marrow metastasis in AML [45].

A study by Gallazzi et al. on tumor associated circulating NK (TANK cells) in prostate cancer showed that these types of NK cells with CD56^bright^CD9^+^CD49a^+^CXCR4^+^ phenotype are expressing TIM-3 and PD-1 and they also produce proangiogenic factors and induce CXCL8, intercellular adhesion molecule 1 (ICAM-1) mRNA expression and vascular cell adhesion protein 1 (VCAM-1) in endothelial cells. Peripheral blood CD14^+^ monocyte-derived macrophages and THP-1 (human monocytic leukemia cell line) are also recruited by the studied NK cells and can be polarized towards proangiogenic M2-like/TAMs [46]. This study may indicate that this phenotype of NK cells can promote AML progression and lead to angiogenesis induction through the above mechanism. However, further investigations on AML patients are needed to clarify this issue.

Acidic and a low-pH microenvironment in BMM damages the NK cells cytotoxicity and altering their metabolic signatures [47]. NK cells downregulated expressing activating receptors such as NKp44, NKp30, NKp46, NKG2D, and granzyme B and perforin in hypoxia. NK cell metabolism was profoundly affected by TGF-β. A study showed that Galunisertib as a clinical TGF-β receptor-I inhibitor, caused keeping the CD16 and NKG2A expression on NK cells, improved NK cell dysfunction and delayed tumor growth in leukemia models. As recognized by Viel et al., TGF-β has an inhibitory impact in the mTOR activity in IL-5-stimulated NK cells that is impaired NK cells development and differentiation in vivo [48].

The pathogenic and prognostic role played by VEGF and its receptor (VEGFR) in AML makes them important anti-cancer therapy targets. In patients with AML, new data suggest that lymphangiogenic growth factors, including VEGF-C and its receptor, VEGFR-3, are closely linked to poor prognosis, the proliferation, survival of leukemic cells, and cancer cell infiltrate dissemination via lymphatic or blood vessels [49]. Taken together, NK cells' ICs or produced components by them can be noticed as a therapeutic targeted factors in AML cases which contribute to poor clinical outcomes and AML progression.

### Dendritic cells (DCs)

Studies show that in AML patients both subgroups of dendritic cells (DCs), namely myeloid (mDCs) and plasmacytoid (pDCs), undergo genetic changes. DCs derived from AML patients (AML-DC) originated from the leukemic cells clone and may exhibit leukemic antigens [50]. The DCs could contribute to cancer growth through IL-1β production. IL-1β is the main cytokine in inflammation-related myeloid disorders. This is expressed by myeloid DCs, supporting cellular expansion and progression of disease [51]. In addition, DCs contribute to tumor progression by producing IL-10 and tolerogenic signals [50]. Inhibitory ICs/ligands expressed by bone marrow DCs are PD-1 [52], PD-L1 [52, 53], PD-L2 [53].

Several functional alterations cause the escape of leukemic cells from the immune system [54]. Malignant cells productions, like TGF-β, VEGF, and IL-10 can trigger the DCs dysfunction, which leads to the ineffective presence of tumor-associated antigens in lymphocytes. Besides, due to immature DCs in leukemia patients, suppressive or regulatory T cells can be induced and the anti-leukemia immune response quality might be impaired. M. Mohty et. al have demonstrated that the co-stimulatory molecules CD80 (B7-1) and CD86 (B7-2) expression decreased in AML whose interaction with CD28 is essential for triggering expansion, activation, and differentiation of T cell. The reduction of HLA-DR on the leukemic pDC subset is another functional change that affects their stimulatory potential via the CD40 pathway [55, 56]. Furthermore, the infiltration of pDCs in the skin and lymphoid organs of patients with myeloid malignancies leads to a poor prognosis condition named tumor-forming pDCs (TF-pDCs). PDCs are the main producers of type I IFN known as IFN-α after microbial stimulation. An investigation tested the pDCs capacity to produce IFN-α after stimulation with HSV in leukemic patients. The TF-PDCs secreted less IFN-I in contrast to healthy donors, which causes the protection of leukemic cells from immune responses [54]. Overall, various DC types appear to affect clinical outcomes in AML patients and they should be noticed as important cells in AML progression and impaired immune response in leukemia.

### Myeloid-derived suppressor cells (MDSCs)

MDSCs are innate immune cells obtained from bone marrow, which have a suppressor role in adaptive and innate immune responses [57]. MDSCs accumulation has been observed in several myeloid disorders, including myelodysplastic syndromes (MDS) and AML. However, their role in suppressing antigen-specific T cells should be explored [23].

In solid tumors, MDSCs generate a cellular microenvironment in which transformed cells proliferate, acquire new mutations, and escape host immune surveillance. In humans, still, their phenotypic description is controversial because there are no clearly defined agreed markers. It is possible to divide MDSCs into monocytic (CD33^+^/CD14^+^/HLA-DR^low^) and granulocytic (CD66b^+^/CD33^+^/CD14^+^/CD15^+^) subtypes [58]. Recently, it has been found that MDSCs (CD33^+^/CD11b^+^/HLA-DR^low/neg^) in the BM are markedly increased in adult AML patients [59]. It has been indicated that TIM-3, with the expression on DCs and T cells, can suppress immune responses indirectly by stimulating MDSCs proliferation. Additionally, MDSCs at the leukemia site is differentiated into TAMs [60]. High PD-L1 levels are expressed by tumor-infiltrating MDSCs [61].

Due to the activation of HIF‐1α‐dependent increase of arginine (Arg) activity and nitric oxide (NO) generation, tumor-derived MDSCs have recently been shown to be highly immunosuppressive. Under hypoxia, the tumor MDSCs upregulate PD-L1 expression, which improves MDSC-mediated T cell tolerance. HIF-1α is therefore an important regulator of PD-L1 mRNA and protein expression. Signaling by G-CSF, GM-CSF, and tumor-derived cytokines via STAT5 and STAT3 induces the development of lipid transporters and improves the absorption of lipids that are available in high amounts in the tumor microenvironment (TME). Oxidative metabolism is enhanced by intracellular lipids and the MDSC immunosuppressive activity is increased. The immunosuppressive effect of MDSC is inhibited by the STAT5 or STAT3 signaling reduction, or the genetic removal of the fatty acid translocase CD36, resulting in improved CD8^+^ T cell performance and slower tumor development [62]. The MDSCs seem to influence the clinical outcome and possibly can be used as a therapeutic target in AML cases [59].

### leukemia-initiating cells (LICs)

The first leukemia-initiating cells (LICs) were discovered in the CD34^+^CD38^−^ section of AML cells. They are involved in the initiation of human AML in NOD/SCID mice [63]. LICs are thought to drive chemoresistance and relapse in acute leukemias. LICs have many characteristics in common with normal hematopoietic stem cells (HSCs). For survival and proliferation, LICs keep partial dependence on signals originated from the hematopoiesis-regulating BM microenvironment. However, LICs are able to dominate HSCs, occupying the BM microenvironmental niches [64]. Therefore, it is believed that leukemic cells lead to the disruption of healthy BM niches for creating "leukemic" niches.

CD 47, a ligand for signal regulatory protein alpha on DCs and macrophages is expressed by LICs in high levels and it induces macrophage mediated phagocytosis inhibition for LICs. AML LICs with a lack of expression of NKG2L can escape from NK-cell-mediated lysis. LICs have increased expression of CD200 in comparison to normal HSCs and this marker is positively associated with apoptosis reduction and inflammatory immune response downregulation in AML cell lines [65]. It is believed that LICs play a significant role in AML development. Thus, their eradication is crucial to achieving effective treatment. Taussig et al. have shown that using anti-CD38 antibodies could eradicate some LICs from immunodeficient mice via immune clearance [66]. As a result of the abrogation of this effect, they found that the CD34^+^CD38^+^ fraction of seven AML samples initiated leukemia in immunodeficient animals. Additionally, some leukemias did not contain any LICs in the CD34^+^CD38^−^ fraction. Therefore, LICs appear to have a more heterogeneous phenotype than what is described by the original studies [66]. As a result of LIC proliferation, the hypoxic niche expands [67]. LICs may produce chemicals like SCF, enter niches, and exploit the normal hemostatic mechanism to promote proliferation and boost self-renewal. Since the spreading of LICs leads to the expansion of hypoxic niches, to overcome chemoresistance, growth factors and other adhesion receptor signals of these cells can be targeted [67].

Consequently, based on the pivotal functions of LICs in the advancement of AML and the development of chemoresistance, it is imperative that the significance of these cells on leukemic cells be comprehensively elucidated in forthcoming investigations and regarded as plausible targeted therapies.

### Mesenchymal stem cells (MSCs)

Most hematopoietic cells require a direct link with stromal cells for their differentiation and growth. Stromal cells have been proven to generate different kinds of growth factors that are required for cell differentiation and growth [68]. In the case of leukemia, the interaction of leukemic cells with stromal cells results in detrimental changes in leukemic cells which could be a serious issue in the treatment of leukemia. Several studies have shown that stromal cells are protective of leukemic cells by preventing apoptosis and leading to tumor invasion by angiogenesis [69–71]. Mesenchymal stem cells (MSCs) that mostly surround sinusoidal and arterial vessels constitute a heterogeneous population of non-hematopoietic stem cells. It has been demonstrated that MSCs can generate a broad range of mature mesenchymal cell types in the stromal microenvironment, like chondrocytes, osteoblasts, adipocytes, as well as fibroblast-like cells.

In a recent in vitro study, Garrido SM and colleagues demonstrated that a human bone marrow stromal cell line, HS-5, enhanced AML cell survival and weakened chemotherapy-induced cell death [72]. Stromal cell-induced proliferation may be related with the progress of the minimal residual disease (MRD) that is a prognostic factor in leukemia [73]. Various research works have proposed the potential role of anti-apoptotic proteins in stromal-supported hematopoietic cell survival. M Konopleva et al. have investigated the impact of MS-5 stromal cells on AML cell survival in the myeloid leukemia cell lines NB-4 and HL-60 and primary AML samples. Consequently, they have revealed that elevating Bcl-XL and Bcl-2 levels mediate the anti-apoptotic impact of MS-5 stromal cells on primary AML cells and HL-60 cells, and the elevated expression level of Bcl-2 in stromal-supported AML blasts in vitro is associated with chemotherapy resistance in vivo [74].

Hematologic malignancies, as well as solid tumors, need vascular support that is enhanced by MSCs. These cells are supporting tumor vasculature through differentiation into endothelial cells or pericytes or the secretion of proangiogenic factors and in this manner have a crucial role in the angiogenesis of hematologic malignancies and solid tumors [75, 76]. Rodrigues Lopes et al. have reported that the MSC cytokine pattern in AML patients includes elevated expression levels of CXCL12, VEGFA, PGE2, IL-1β, indoleamine 2,3-dioxygenase (IDO), IL-32, and IL-6, and reduced expression of IL-10. IL-32 supports stromal proliferation and chemotaxis [77]. On the other hand, MSCs derived from AML patients release CXCL8 which is a pro-inflammatory chemokine that can increase the proliferation and survival of AML blasts through the phosphoinositide-3-kinase (PI3K)/AKT pathway. Moreover, Yuanye Li et al. have concluded that CXCL8 levels is significantly higher in plasma samples from patients with AML in comparison with normal individuals [78]. These results indicate that MSCs could be the main producers of CXCL8 in the AML BMM. Some stroma-released chemokines facilitate the recruitment of macrophages to tumor tissues. As an example, there is an association between the C–C motif chemokine ligand 2 (CCL2) elevation and increased macrophage infiltration and poor prognosis of cancers [79]. Moreover, higher CCL2 plasma levels have been shown in patients with AML [80]. MSCs also contribute to the regulation of HSCs function through the production of angiopoietin-1 (Ang-1) and SCF [81]. The MSCs secrete matrix proteins and cytokines such as VEGF and platelet-derived growth factor (PDGF) that elevates proliferation and supports vasculogenesis [82]. Another angiogenic soluble factor, such as fibroblast growth factor-1 (FGF-1) is produced by MSCs [83, 84]. Therefore, MSCs could modify the expression profile of angiogenesis-related chemokines in AML cell lines [85].

Collectively, MSCs as a group of AML BMM cells, directly or by contributing to other cells can exert a major role in AML progression and poor clinical outcomes. Hence, more studies are required for finding therapeutic targets for the mentioned cells in patients with AML.

### Osteoblasts and osteoclasts

Recent studies suggest that osteoblasts contribute to pre-leukemic conditions in mice. Osteoblasts in the endosteal niche are essential for HSC long-term persistence and bone marrow retention [9]. Investigations have shown that an activating mutation of β-catenin in mouse osteoblasts influences the differentiation potential of lymphoid and myeloid progenitors contributing to AML development with common chromosomal abnormalities and cell-autonomous progression. Furthermore, studies have confirmed that AML is induced by defective niche signals within the bone marrow osteoblasts.

Also, research has indicated that myeloid malignancies may be induced by osteolineage cells [86]. Through signaling pathways like Ang-1/Tie-2, Jagged-1/Notch, and TPO/MPL, osteoblasts limit HSC differentiation and promote self-renewal in order to preserve the HSC pool [87]. Furthermore, osteoblasts produce some extracellular proteins and hematological cytokines such as G-CSF, Osteopontin (OPN), interleukins, type I collagen, stromal cell-derived factor-1 (SDF-1), and osteocalcin (OCN) [88, 89]. OPN (early T-lymphocyte activation-1) is a protein produced by hematopoietic cells and osteoblasts in the bone marrow. The integrin-α9 pathway increases metastasis when OPN is overexpressed as a lymphangiogenic factor. In AML, OPN serves as a prognostic factor for survival [90]. OPN and SDF-1 may encourage CXCR4^+^ leukemia stem cells to migrate to the osteoblastic niche. SDF-1 interacts with its receptor on leukemic progenitor cells, allowing them to settle in the BMM [89].

In the peripheral circulation and BM, the associated VEGFs, cytokines, and receptors are expressed on AML blasts in vascular osteoblast niches [90]. Following chemotherapy, LSCs settle in the endosteal area. According to Ishikawa et al. VEGF-A from sinusoidal endothelium, MSCs, and osteoblasts in the endosteal niche are implicated in AML LSC niche-related regressions since a protective niche is required by resistant leukemic blasts for spreading [91]. The BM cytokine VEGF-A is a representative cytokine for predicting poor outcomes and defining AML subtypes [90]. Pro-angiogenic compounds, such as VEGF, CXCL8, FGF, and MMPs, are produced by BMSCs and osteoclasts, and they are frequently induced by the interaction between AML cells and BMSCs, as well as by genetic or transcriptional alterations. Consequently, these cells may have a role in the AML pathogenesis in humans and provide a valuable attitude for future therapeutic approaches.

### Adipocytes

MSC-derived adipose tissue in the BM, as an extra-medullary storage place for normal HSCs, is called BM adipose tissue (BMAT) [87]. Studies have shown that only small adipocytes in BM, and not total adipocytes are associated with a poor prognosis for AML patients [92].

Adipocytes in the BM are generally considered negative regulators of hematopoiesis. In addition, by suppressing leukemic adipocytes in the BM, imbalanced regulation of hematopoietic stem cells and progenitor cells is established, resulting in impaired myelo-erythroid maturation [93]. In BM of AML patients, adipogenesis is one of the reasons that can result in aplastic anemia . Zhao et al. have found that adipocytes in different BM compartments in mice play different roles. For example, adipocytes maintain stem cells by secreting SCF in long bones but suppress hematopoiesis in caudal vertebrae. In addition, this study hypothesizes that adipogenesis will act as an emergency response to cytopenia, which will stimulate fast hematopoiesis, emphasizing the significant connection between BMAT and hematopoiesis [94].

Adipocyte remodeling plays an integral role in AML development. The living area of adipocytes is constricted because of the rapid development of leukemic cells in confined bone marrow cavity, triggering a number of adipocyte remodeling events such as morphological alterations and lipolysis [92]. Shafat MS et al. investigated the interactions between adipocytes and leukemic cells and they discovered that, under the influence of AML cells, BMAT was switched into a lipolytic phase, with subsequent production of free fatty acids to provide nutrients for leukemic cells [95]. Growth differentiation factor-15 (GDF-15), which is produced by leukemic cells, has been shown to help small adipocytes differentiate from larger adipocytes by releasing into the BM cavity. Small adipocytes induced by GDF-15 displayed enhanced lipolytic activity with the elevated expression of lipolytic genes, e.g., ATGL and HSL. As a result of lipolysis, higher amounts of free fatty acids were generated, which provided many of the energy requirements of leukemic cells [96]. Additionally, adipocytes produce a large amount of adiponectin in the caloric restriction condition, such as cancer therapy. Adiponectin inters AML myeloid cells through AdipoR1and causes activation of AMPK that leads to activation of heat shock protein 90 (HSP90). Upregulation of HSP90 and AMPK-activated Co-chaperon immunophilins protein in cytoplasm causes survival of AML cells [97]. Furthermore, adipocytes cause short survival of AML cells by FABP4 production that stimulates overexpression of IL-6 and activation of NF-ĸB in AML cells. Interestingly, according to the reports, secreted FABP4 causes the AML cell proliferation in fat mice [98]. Thus, the disruption of AML-adipocyte interactions may serve as a new targeted therapeutic approach for AML patients.

### Cancer-associated fibroblasts (CAFs)

Cancer-associated fibroblasts (CAFs) have an essential role in migration and survival of leukemic cell [99, 100]. As revealed by Zhai et al., numerous functional CAFs are located in the BM of patients with AML. CAFs protect the AML cells from chemotherapy via growth differentiation factor 15 (GDF15) secretion [101]. Co-culture of AML blasts with fibroblast lines (Hs27 and HLF1) and normal BMSCs revealed the importance of fibroblasts in AML development. AML cells displayed decreased proliferation, a decreased capacity to escape apoptosis, and lower synthesis of CXCL8 in the absence of fibroblasts [102].

CD73, an immune checkpoint that also is an ecto-5′-nucleotidase (NT5E), generates adenosine (ADO), which exerts its immune suppressor activity through the A2A receptor [103]. According to recent studies, it has been found that CD73 expression was increased in AML patients with NPM1 gene mutation. The authors have suggested that the combination of clinicopathologic features, CD73 expression, and NPM1 gene expression could be helpful as a prognostic marker and a guide for the development of relevant therapeutic approaches [104]. Investigations have reported that CAF-CD38 promotes the expression of VEGF-A, HGF, FGF-2, CXCL12, and MMP-9 proteins, which are associated with angiogenesis and metastasis [105]. Thus, we suggest that it seems to be critical to focus on the A2B-mediated ADO-CAF-CD73 feed-forward circuit as well as A2A-mediated immune suppression for effective MEDICAL approaches in AML patients.

AML blasts and myeloid leukemia cell lines, including KGI, HL-60, and K562, stick to fibroblasts in the BM. VCAM-1 is a cell membrane protein found on BM fibroblasts, and its expression is influenced by cytokines, e.g., TNF-α, IL-1, and IL-4 [106]. As shown by researchers, AML blasts can manipulate fibroblasts. The result is that AML reshapes the microenvironment in a manner supporting the proliferation and survival of AML blasts [107]. This implies that fibroblasts and malignant blasts must interact in order for cancer cells to survive and migrate. EEMMPRIN (CD147) is a glycoprotein found on the human tumor cell surface that induces stromal cells and tumor cells for generating more MMPs, leading to ECM destruction and increased tumor growth and metastasis [108–110]. CD147 has been found to stimulate MMP2 release from fibroblasts in various tumor cell types [107]. In AML, researchers have discovered that VEGF and EMMPRIN co-expression indicates a poor prognosis [111]. Furthermore, a lack of CD147 in the AML cell line U937 caused apoptosis, repressed cell proliferation, and improved the efficacy of the cytotoxic drug Adriamycin [112]. Thus, CD147 is a potential therapeutic and prognostic target for AML. Together, CAFs as potential therapeutic targets for AML cases exert a significant role in AML progression. While many advances have been made in in-vitro studies, more in-vivo surveys are required for understanding its behavior in cross-talk with leukemic and other BMM cells that may contribute to AML progression and poor clinical outcomes.

### Endothelial cells

Many cytokines involved in the differentiation and proliferation of hematopoietic progenitors have been shown to be induced by endothelial cells [113]. Leukemic cells release cytokines, particularly IL-1β and TNF-α, which activate endothelial cells when they come into direct contact with their adhesion receptors. Therefore, leukemic cells promote their adherence to the endothelium of blood vessels [114]. ICAM-1 (CD54), VCAM-1, and P- and E-selectin (CD62P and CD62E) are binding partners on BM endothelial cells [115]. E-and P-selectin regulates HSC rolling on the endothelium, then, HSC can stick to the endothelium via integrins and move to the BM stroma. Furthermore, an important ligand of CD44 on HSCs is hyaluronic acid (HA), which is secreted by endothelial cells [99]. Endothelial cells have been demonstrated to enhance leukemic proliferation by secreting cytokines, like G-CSF, IL-3, IL-6, nitric oxide (NO), and GM-CSF [67]. The Notch/Dll4 pathway promotes angiogenesis via interactions between AML and endothelial cells [116]. It has been shown that patients with AML have high VEGF levels, which lead to angiogenesis and reduced apoptosis. Further, culturing endothelial cells with VEGF increases endothelial cell production of GM-CSF, a factor known to promote AML cell proliferation [117].

### Schwann cells

Instead of being categorized only as supporting cells, it has been shown that neuroglial cells have a role in managing the size of the HSCs pool. By producing TGF-β, Schwann cells provide a quiescence signal to HSCs in the BM niche. It has been shown that TGF-β-producing cell populations are reduced by sympathetic nerve denervation, which results in a fast elimination of HSCs from BM [87]. Glial cells are considered part of the BM niche and sustain hibernation of HSC by controlling the latent TGF-β activation [118]. Also, myeloproliferative neoplasms (MPN) patients show fewer sympathetic nerve fibers in the BM that support Schwann cells and Nestin^+^ MSCs. This is due to IL-1, released by mutant HSCs and promotes their development. Using β3-adrenergic agonists as the treatment stopped MPN development and halted the loss of Nestin^+^ MSCs [99].

### T lymphocytes (exhausted, regulatory, and γδ T cells)

In AML cell states, monocyte-like AML cells exhibited immunomodulatory activity and suppressed T-cell activation. Several inhibitory T-cell ligands (including Gal-9, PD-L1, CD112, CD155, CD86) are overexpressed in AML blasts, impairing T- and NK-cell function [119]. Asgarian-Omran and Taghiloo in their study described the role of co-inhibitory pathways in AML in detail [23]. One of the best-known AML ligands for ICs is the PD-L1, which can cause the exhaustion of T cells with its co-inhibitory signal when it is recognized by PD-1. In addition, PD-1/PD-L1 promotes regulatory T cell expansion (Tregs). Another ICs named TIM-3, as a recognized IC, is found on effector T and NK cells and also being overexpressed on AML blasts. Some research has shown that self-renewal is promoted by TIM-3/Galectin-9 (Gal9) signaling via NF-κB and β-catenin signaling and pro-inflammatory cytokines are reduced, leading to NK and T cell dysfunctions. As a result, Gal9, as a TIM-3 ligand, appears to be essential to maintaining LSCs through an autocrine loop [21, 120]. Leukemia decline in patients with AML following allogeneic stem cell transplantation is related to high TIM-3^+^ PD-1^+^ T cell levels [121]. According to earlier research works, CD34 and TIM-3 were significantly elevated in all AML groups and cell lines [122–126]. Recent studies have also demonstrated that high levels of inhibitory receptors, CTLA-4 and LAG-3, on AML blasts are related to poor prognosis [127].

Le Dieu et al. apperceived an increase in the total number of peripheral blood T cells and CD3^+^ CD56^+^ cells (T lymphocytes with NK activity) of newly detected patients of AML in comparison to age-matched healthy controls. Their data indicated a disorder in the formation of immune synapses by T cells, Nevertheless, pointed normal levels in BM [128]. Also, Lim Sh et al. showed that the percentage of CD3^+^ lymphocytes in AML patients, whether active or fully recovered, was increased. However, when the cytotoxic function of the cells was analyzed in this case, all AML patients, whether with active disease or full recovery, had dysfunctional lytic cells [129]. This discrepancy is probably due to the suppressive effect of leukemia myeloblasts. AML blasts do this by making change in the cytokine environment and release soluble factors, like indoleamine 2,3-dioxygenase-1 (IDO1), reactive oxygen species (ROS), extracellular vehicles (EVs), and arginase II (ArgII) [130]. It has been indicated that high Arg II levels in plasma of patients with AML damage T cell proliferation, polarizing monocytes toward an immunosuppressive M2-like phenotype [131].

AML blasts induce T-cell apoptosis and exhaustion and expand regulatory T cells (Tregs) and MDSCs [132]. Shenghui et al. have indicated that there is an association between the elevated frequency of CD4^+^ CD25^+^ CD127^low/ −^ Tregs in AML and poor prognosis. The presence of more immunosuppressive BM-resident Tregs than those detected in peripheral blood (PB), further supports the notion that AML niches are composed of many inhibitory layers [133]. T-cell exhaustion is described as a state of T cell dysfunction resulting in the increase of inhibitory receptors (PD-1, CD244, CD160, LAG-3, TIM-3) with poor effector function (hyperproliferation, diminished cytotoxicity, reduced cytokine generation), and progressive loss of T cell function in cases with AML [134]. The production of VEGF, TGF-β, IL-6, IL-10, and other inhibitory cytokines activate STAT3 signaling, inhibit effector CD8^+^ T cell differentiation and induce exhaustion in favor of the generation of stem-cell like memory T (TSCM). Furthermore, the overexpression of inhibitors of DNA-binding/ differentiation (ID) transcriptional genes and STAT3 signaling in mature CD8^+^ T cells enhances the production of TSCM cells [135]. There has been an association between Treg enrichment in the AML niche and immune-suppressive factors released by AML blasts, e.g., IL-10, IL-35, TGF-ß, and IDO1 [136–138]. Specially, it has been found that there is an association between IDO1 and a poor prognosis. By increasing IDO1, T-cell proliferation is arrested by the decrease in local tryptophan concentrations and the gathering of toxic metabolites of tryptophan. In addition, the metabolites of tryptophan, such as L-kynurenine, impede antigen-specific T cell proliferation, causing them to undergo apoptosis [139]. Moreover, AML blasts expressing increased levels of inducible nitric oxide synthase (iNOS) are associated with decreased proliferation of T cells, and an elevation in T-regs [140]. Increased frequencies of Tregs and their vigorous suppressive activity in AML patients compared to healthy controls have been proved previously. The TNFR2^+^ Tregs signify a very effective Treg subset. The highly inhibitory role of TNFR2^+^ Tregs in the human tumor microenvironment (TME) has shown that the increased levels of TNFR2^+^ Tregs denote their robust suppressive capacity [141]. Accordingly, as shown by earlier research works, TNF-α–TNFR2 interaction is crucial to activate and expand functional Tregs [142]. Additionally, levels of circulating Tregs CD4^+^CD25^+^, the level of TNFR2, and CD4^+^CD25^high^ T cells are higher in patients with new diagnosis of AML than in healthy individuals or complete remission patients. The previous findings show that the TNFR2 expression frequency on peripheral blood CD4 +  T cells can be an easily available and novel marker for predicting clinical outcomes or monitoring AML patients' progress [143]. Additionally, Foxp3 as a transcriptional factor has a significant role in differentiation and cellular function of Treg cells. This factor has four functional domains that its DNA binding domain is called winged-helix/forkhead (FKH). In a study by J.-H. Park et al. synthetic FKH domain that was carrying to the nucleus via Hph-1-PTD suppressed Treg cells and rebalanced TME through downregulation of CTLA-4 and IL-10 and upregulation of IFN-γ and IL-2 in Treg cells [144]. In another study, this factor observed in about 95 percent of CD4^+^ CD25^+^ cells that was associated with a poor prognosis [145].

Interestingly, STAT5 is an important factor in Treg cells [146]. Since Treg cells contain a high content of CD25, they are sensitive to even a low dosage of IL-2 compared to conventional T cells. This high sensitivity leads to phosphorylation of STAT5 and further causes the proliferation of Treg cells [147]. Significantly, leukemic cells (both CML and AML) release Rab27a dependent 4-1BB containing endovesicles that upregulate STAT5 activity in Treg cells that leads to overexpression of effector/tumor Treg markers such as CD39, CCR4, TIGIT, TNFR2, CD30, and CCR8 via expression of Foxp3 [148].

CD155 as an adhesion marker is expressed on normal organs such as kidney, liver, and lung in a low level. But in several cancers its overexpression leads to metastasis and proliferation of cancer cells. Interestingly, interaction of CD155 and its ligand DNAX-associated molecule-1 (DNAM-1) on cytosolic T cells and NK cells empowers anti-tumor action of these cells in the initial phase of cancer. But in the late phase of cancer, causes progression of cancer. For instance, CD155 causes down-modulation of DNAM-1 in NK cells and disturbs the cellular function of these cells in AML [149]. On the other hand, DNAM-1 can express on AML leukemic cells. According to studies by A. Chashchina et al., interaction of DNAM-1 and its ligands (CD155 and CD226) causes production of modulatory cytokines, e.g., TNF-α, IL-6, IL-8, and IL-10 that results in proliferation and survival of AML cells [150]. In the following, ITIM domain (TIGIT) is another receptor of CD155 that is co-inhibitory expressed on NK, Treg, and CD8^+^ T cells. Notably, Kong et al. showed that expression of TIGIT on CD8^+^ T cells has a direct relation with the amplification of exhaustion markers, like CD160, PD-1, 2B4 on CD8^+^ T cells and leads to exhaustion of these cells [151].

CD200 as a negative antitumor immunity modulator that is related to poor prognosis in individuals with AML, with the expression on tumor cells in a high level. Interestingly, A. Memarian et al. have blocked CD200/CD200R in AML cells and revealed that this blockage decreased Foxp3 in Treg cells. These data show CD200 activity in AML has a direct effect on Foxp3 level in Treg cells [152].

One of the unconventional T cell subsets is gamma-delta T (γδ T) cells. Circulating γδ T cells have a substantial role in operating the process of both recognizing and destroying abnormal cells as a part of the immune response. As shown by previous research findings, stimulated γδ T cells have the capability to recognize and kill AML blasts [153]. Interestingly, Hoeres Ti et al. have indicated that lower signaling of TCR and IL-2 receptor and adequate expression, function, and involvement of PD-1 receptor by its ligand improves the anti-tumor functions of γδ T cells [154]. Previous research showed that a lack of ICs signaling could stimulate IL-17-driven γδ T cell immunity as pro-inflammatory cytokines, which induce different ICRs to express on the γδ T cell surface [155, 156]. Importantly, CD73 is expressed on several cells in TME, such as endothelial cells, Treg cells, stromal cells, and tumor cells. This CD marker facilitates TME for tumor growth via catabolizing of AMP to adenosine that suppresses effectory T cells. Interestingly, expression of this CD marker in AML patient has controversial effects. Although some studies have reported the association between the CD73 expression and poor prognosis. Other studies say expression of this CD marker on CD8^+^ T cells has had promising outcomes and a decline in the expression of suppressive markers on CD8^+^ T cells such as PD-1, TIGIT, and LAG-3 [11]. On the other hand, it has been revealed that expression of CD73 along with CD39 on үδ T cells has antitumor immunity activity. Accordingly, As shown by Brauneck et al., үδ T cells cause the expression of high level of CD39 compared to CD4^+^ and CD8^+^ cells. This study indicates that expression of CD39 causes immunosuppressive conditions through the recruitment of MDSCs. Also, expression of CD39 in үδ T cells elevates Foxp3 in these cells that suppresses αβ T cells [157].

CD38 is another ectoenzyme that catabolizes NAD^+^ and NADP along with CD31. CD38 is expressed on several cells such as lymphoid, myeloid, RBC, and platelet in normal condition. Significantly, J. Naik et al. have reported that CD38 is expressed on AML blasts and Treg cells adequately in AML patients that can be targeted in several therapeutic strategies such as CAR T cell therapy and blockage of CD38 via monoclonal antibodies [158].

Th17 is one of the subgroups of CD4^+^ T cells with some crucial activities in inflammation, autoimmunity, graft-versus-host disease (GVHD), and cancer progression [159–161]. IL-17 is the most important cytokine that is secreted from Th17 cells that has a critical role in the angiogenesis, proliferation, and metastasis of several cancers. Accordingly, Han et al. have proven that the level of Th17 cells have been increased frequently in bone marrow and peripheral blood of patients with AML and cause U937 AML cell proliferation through secretion of IL-17. Also, this study suggests increasing of Th17 compared to Th1 is related to poor prognosis in AML [160].

In addition, with the elevated FOXP3, IL-6, and IL-17 levels in human primary cord blood-derived T cells, exosomes derived from CML cells can influence the fate of T cells [162].

Therefore, T cells with their important role in connection with tumor and leukemic cells, should be considered as vital cells in tumor suppression and even though progression. By empowering them to become active forms and manipulating their inhibitory immune checkpoints, it is suggested to promote anti-cancer immunity in AML patients.

### B lymphocytes

During the survival and growth of acute myeloid cells, the leukemic cells interact with stromal cells that secrete or express superficial growth and survival factors. By using an animal model, Sipkins et al. have demonstrated that functional CXCR4 is required for AML cells to home in BMM [163]. In addition, the CXCR4 ligand (CXCL12) is essential for normal B-cell development because it retains pre-B cells in close proximity to supportive stroma cells within the hematopoietic microenvironment [164]. Leukemic cells from B cell chronic lymphocytic leukemia (B-CLL) patients, B-cell acute lymphoblastic leukemia (B-ALL), and AML, significantly express CXCR4. Since the partially hypoxic BM environment upregulates the CXCR4 and CXCL12 expression [165], there seems to be a connection between these signaling paths of the B cells and AML blasts. On the other hand, some of the previous studies have indicated that MMPs expression is related to the potential metastatic event of various human tumors, and the modulation of MMPs affects some transcriptional factors, particularly NF-κB and STAT families leads to increased levels of VEGF transcriptional regulators [166, 167]. It has been shown that AML blasts and B cells express VEGF receptors in both BM and the peripheral circulation which can accelerate their migration [168]. Thus, the expression of some common signal pathways between B cells and AML blasts can contribute to AML pathogenesis which needs further investigation.

## Immune checkpoints’ roles in AML angiogenesis, metastasis, and cell proliferation

Immune checkpoints are groups of co-stimulatory and inhibitory receptors that positively or negatively regulate the immune system, homeostasis of the immune system, and avoiding autoimmunity depend on them. Cancerous cells escape immunity by disrupting immune checkpoints intelligently. Immune checkpoint blockers act by activating co-stimulatory signals and blocking inhibitory signals. The discovery of novel immune checkpoint molecules as potential cancer therapeutics resulted in a substantial increase in studies involving novel therapeutic agents [169]. Here, we will highlight the functional role of co-stimulatory and inhibitory ICs in AML by focusing on signaling mechanisms that cause angiogenesis, metastasis, and cell proliferation in this malignancy. We propose that the import of these ICs on leukemic cells be thoroughly expounded upon in future inquiries and considered as promising multi-targeted treatments in AML.

### Co-stimulatory immune checkpoints

Co-stimulatory immune checkpoints can enhance immune responses against cancerous cells, and cancerous cells initiate tumorigenesis through these stimulatory pathways suppression [170]. In the following sections, we have reported some of the most notable studies that investigated the modulatory effect of immune checkpoints on AML:

#### CD40/CD 40-L

In AML patients, CD40 expression is associated with poor prognosis, and it is shown to have a direct impact on cancerous cells [171]. A significant correlation between poor outcome and CD40 expression by blast cells was observed when analyzing the overall survival of a large group of AML patients [172]. According to a study by Donatella Aldinucci et al. CD40 activation stimulates the proliferation of various types of cells including CD34^+^ cord blood progenitor cells, B cell precursors, as well as tumor cells from varied origins and also, CD40-L enhances the self-renewability of AML cells [171]. Soluble human CD40-L (sCD40L) inhibits apoptosis by decreasing APO2.7 and annexin-V protein binding and by increasing Bcl-xL, a natural anti-apoptotic molecule in the absence of Bcl-2 and Bax proteins [171]. CD40-CD40L interactions also promote leukemic growth by simultaneously promoting close cell contact and auto-stimulatory soluble factors like GM-CSF production [173]. Based on studies, there is a certain degree of overlap between CD30L and CD40 expression in AML. CD30L expression by AML blasts correlates with IL-4 receptor expression and the expansion of helper T cell 2 (Th2) cells. IL-4 is able to enhance in vitro proliferation of leukemic blasts [174]. Therefore, CD40 engagement by its ligand on AML blasts triggers pleiotropic responses involving proliferation, survival from apoptosis, self-renewal capability, and production of growth-promoting cytokines. Thus, CD40 inhibition by treatment with specific inhibitors such as monoclonal antibodies, CDX-1140, and Dacetuzumab can inhibit the pathways induced by CD40 in AML patients and improve treatment outcomes [175–177].

#### CD80, CD86

CD80 (B7-1) and CD86 (B7-2) are ligands of the B7 family that comprise structurally related, cell surface proteins that modulate immune responses through the delivery of co-stimulatory signals. Both CD80 and CD86 are expressed innately in many hematologic malignancies, but they are rare in acute leukemia [178]. APC activity and T-cell priming are both enhanced after follicular lymphoma (FL) cells are activated in vitro by up-regulating CD80/CD86 and other stimulatory and adhesion molecules [179]. The CD80 or CD86 binding to its receptor (CD28) triggers the PI3K/AKT/mTOR signaling pathway leading to IFN-γ, IL-2, and BCL-XL production. However, the expression of these co-stimulatory molecules alone is clearly insufficient for effective anti-tumor immunity, since even malignancies that produce high levels of these molecules inevitably progress without treatment [180]. Studies suggest that CD80 might be influencing the progression and metastasis of breast cancer by regulating the innate immune system [181]. While the potential role of CD80 in the metastasis of AML has not been demonstrated. Hence, more investigations are required to understand the possible role of CD80 in AML metastasis.

An in-vivo experiment in mice demonstrated that cytosine arabinoside (cytarabine) reduced the expression of PD-1 on leukemic cells while enhancing the expression of CD80 and CD86, making the leukemic cells more susceptible to killing by T lymphocytes. A total of 14 of 21 human AML sample cultures were induced to express CD80 or CD86 by cytarabine [182].

### Inhibitory immune checkpoints

Inhibitory pathways of immune checkpoints suppress T cell activation and duration of immune responses, as well as inflammation, tolerance, and homeostasis and by hijacking these checkpoints, tumors disable the immune system [183]. In the following parts, we have reported some of them in AML:

#### CTLA-4

CTLA-4 (cytotoxic T-lymphocyte-associated antigen-4) or CD152 is an inhibitory checkpoint-marker that competes with CD28, binding CD80/CD86 on leukemic blasts and it has been shown to be upregulated in primary AML samples and poor prognosis is associated with it, especially when expressed concurrently with PD-L1 and PD-L2 on leukemic cells [184]. Through activating CTLA-4, NF-ĸB signaling is blocked and IL-2 production is inhibited, so anti-tumor immune responses by tumor cytotoxic T lymphocytes and NK cells become limited [185]. A phosphorylated YVKM motif on CTLA-4 recruits tyrosine phosphatase SHP-2 (SYP, PTP-1D), and this interaction is involved in the inhibition of TCR signaling and also PI3K/AKT/mTOR signaling for activated CD28. There is also a link between CTLA-4 and the promotion of cytokine transforming growth factor-β (TGF-β) production [180, 186]. Studies have shown that the CTLA-4 impediment enhances the potency of AML-derived DCs and results in potent T cell responses against AML cells [187]. Thus, anti-CTLA-4 monoclonal antibodies, such as ipilimumab, indicated beneficial effects against AML cells [188, 189].

### Programmed cell death 1 protein (PD-1)

PD-1 is a T cell immune checkpoint protein inhibiting cellular activation when it binds to the ligands PD-L1 and PD-L2 [190]. As a result of the interaction between PD-1 and PD-L1 on T cells, activation of PI3K and phosphorylation of Zap70 is inhibited, eventually reducing TCR signaling, CD28-mediated co-stimulation, AP-1, and NF-κB activation, and IL-2 production. The overexpression of PD-L1 causes evasion of the host immune system by cancer cells [191]. According to several studies, PD-L1 and PD-1 are increased in the AML hematopoietic microenvironment [190, 192]. Through available data, binding PD-1 to PD-L1 on AML cell lines can increase glycolysis metabolism through AKT/mTOR/HIF-1α signaling and enhance tumor cell invasion, metastasis, and AML cell proliferation [193]. PD-L1 is expressed in varying amounts on AML patient blast cells and it could prompt T cell inactivation and the Treg cell expansion with high CD25, PD-1, and levels. As a result of increasing expression of PD-1 on Treg cells, the immune response is strongly inhibited, and it can have a contribution to the AML progress and PD-1 signaling blockade by anti-PD-L1 antibody shown as a therapeutic approach against AML malignancy [194].

Studies have approved PD-1 antibodies (pembrolizumab, cemiplimab, and nivolumab), as well as PD-L1 antibodies (durvalumab, avelumab, and atezolizumab) for several cancer types, including AML [195].

Sefid et al. integrated an in-silico method and used various computational software/tools to design an immunotoxin containing atezolizumab (anti-PD-L1 antibody molecule) and granzyme B (GrB) molecule. It was indicated that the proposed immunotoxin effectively interact with the PD-1, inducing the GrB part to apply its toxic impacts on the target cells [196].

#### TIM-3

TIM-3 (T cell immunoglobulin and mucin domain 3) plays a key role in various leukocyte functions. It induces pro-inflammatory effects in dendritic cells, while in T cells it mainly suppresses Th1 responses, and it also participates in phagocytosis with macrophages and monocytes [197]. The expression of TIM-3 on malignant cells has been reported in some leukemias such as AML, and its overexpression may contribute to blast proliferation and immune escape [198]. The most famous ligand for TIM-3 in AML is Gal-9 that their interaction leads to phosphorylation of ERK (extracellular signal-regulated kinase) and protein kinase B (PKB, also known as AKT). Through this process, β-catenin pathway activity and NF-κB activation are triggered, which is essential for leukemic cell survival and disease progression [199]. Furthermore, ligation of TIM-3 and Gal-9 in AML cell lines activates PI3K/mTOR pathways, leading to the production of HIF-1α, VEGF, and TNF-α [200]. Hence, TIM-3 mediates the inhibition of immune response in the TME by different mechanisms and plays an important role in the development, invasion, leukemiogenesis, and metastasis of AML [201]. TSR-022 (NCT02817633) and MBG453 (NCT02608268) are two anti-TIM-3 monoclonal antibodies studied in ongoing phase 1/2 trials in solid tumors. Moreover, MBG453 is tested in conjunction with the anti-PD1 antibody PDR001 in patients with AML or MDS (NCT03066648).

#### CD38

CD38, a transmembrane glycoprotein that expresses in myeloid and lymphoid cells with high levels in plasma B-cells, is a promising target for anti-CD38 therapy in Myeloma. Recent studies confirmed that CD38 is expressed on leukemic blasts of some AML patients [202], and a suitable target for adult acute leukemia treatment [158].

Recently, CD38 is known as a novel IC. In vitro and in vivo studies have revealed that CD38 inhibits the proliferation of CD8^+^ T cells, tumor cell killing, and antitumor cytokine secretion. Also, it appears that CD38 blockade is beneficial to reduce anti-PD-L1 resistance and may become a potential therapeutic approach to cancer therapy [203].

According to Liao et al., CD38 is highly expressed in cervical carcinoma tissue and is responsible for dysregulation of the PI3K/AKT signaling pathway. PI3K-Akt-mTOR constitutive activation appears to differ between AML patients, as well as elevated activity within this pathway, which is an adverse prognostic factor in AML [204].

It is not known how CD38 plays a role in the signaling pathways associated with angiogenesis, metastasis, and cell proliferation in AML, and future studies are necessary to better understand this connection. Recently in clinical trials, CD38-specific human monoclonal antibodies have been successfully used to treat patients with multiple myeloma (MM), suggesting CD38 is a viable target for therapy. There is increasing evidence that CD38- Chimeric antigen receptor (CAR) T cells may be an effective and potent immune therapeutic tool, especially in patients with MM who have limited options for chemotherapy [205]. It was shown by Nolan et al. that CD38-CAR NK cell-based therapy may be a potential therapeutic option for patients with CD38-high expressing AML [175].

#### CD73 and CD39

CD73 is a 70-kD protein, glycosylphosphatidylinositol (GPI) anchoring cell surface protein that has a critical role in regulating adenosinergic signaling. Additionally, it has both enzymatic and nonenzymatic activities within cells [206]. CD39 is an integral cell membrane molecule that is Ca2 + and Mg + -dependent, which has a phosphohydrolase function and phosphohydrolases ATP and ADP to produce AMP [207]. A number of factors regulate CD39 expression, including hypoxia, oxidative stress, proinflammatory cytokines, specificity protein 1 (Sp1), and STAT3 [208]. CD39 and CD73 can suppress immune responses via cleaving ATP into adenosine [208]. Recently, CD39 and CD73 have been identified as IC mediators that are widely expressed on stromal and immune cells, as well as tumor cells in the tumor microenvironment (TME). CD73 inhibits anti-tumor immunity by converting AMP to adenosine via the ectonucleotidase activity [209–211]. Also, both CD39 via the purinergic signaling pathway and CD73 via β-catenin/cyclin D1 and EGFR signaling pathways have an important role in tumor growth and metastasis, and cell proliferation [208].

The purinergic signaling pathway includes activating cellular processes via type 2 purinergic receptors (P2) receptors, and increased secretion of ATP/adenosine diphosphate (ADP). Further, adenosine binds to activated P1 receptors and contributes to cell migration, survival, and proliferation [212].

AMP is hydrolyzed by CD73 into adenosine and phosphate by its nucleotidase activity. Adenosine generated by CD73 plays a critical role in tumor immune escape [213]. Aside from its enzymatic role, CD73 is also a signaling and adhesion molecule that can influence cellular interaction with extracellular matrix (ECM) components, including fibronectin and laminin, contributing to cancer invasion and metastasis [214]. Consequently, CD73 serves both enzyme- and non-enzyme-based functions in cancer-associated processes and is not totally independent of each other.

Allard et al. [215] have demonstrated that tumors, as well as host-derived CD73, contribute to tumor angiogenesis. The tumor-derived CD73 stimulates the production of VEGF, while the host-derived CD73 is needed to enhance VEGF-induced angiogenic responses. CD73-deficient mice displayed less angiogenesis in tumors [216]. According to these results, both tumor and host CD73 promote angiogenesis under tumor conditions in-vivo.

Also, it has been shown that CD39 and CD73 are involved in CLL cell proliferation [217]. Recently, studies demonstrated that CD39 plays a key role in a novel AML cell-intrinsic mechanism of cytarabine resistance and that CD39 can be a potential target for a promising therapeutic approach to AML cells sensitized to cytarabine [218, 219].

Several studies investigated antibodies or small molecules, such as APCP, to regulate the CD73 activity in several cancer types that can be investigated in AML cancer [209, 220]. Furthermore, the combination of anti-CD73 with NKG2D-engineered CAR-NK cells achieves a synergistic antitumor effect in CD73^+^ human lung cancer xenograft model [221]. On the other hand, CD73 can induce angiogenesis via VEGF expression, which is implicated in acquired resistance to anti-VEGF therapy. In addition, CD73 levels have been detected in a patient resistant to bevacizumab (an anti-VEGF mAb). Hence, we suggest in this study that CD73 may have potential therapeutic value in overcoming bevacizumab resistance in AML patients, and more research should be done in the future.

#### CD155

CD155 is the poliovirus receptor (PVR) or nectin-like protein 5 because it is a receptor for poliovirus. CD155 interacts and recruits with nectin to facilitate cell migration and enhance cell death. CD155 is an immunoglobulin (Ig)-like molecule with a domain structure composed of three Ig-like loops in the extracellular region, a cytoplasmic region, and a transmembrane region [222]. CD155 is known to have an immunosuppressive function in immune cells. By binding to Ig and ITIM domains (TIGIT) on NK cells and T-cells, it inhibits cytokine production and cell proliferation, leading to the reduction of GATA-binding protein 3 and interferon regulatory factor. In addition, it plays a cytotoxic role via binding to CD226. In addition to its immunological functions, CD155 also functions in cellular differentiation, proliferation, survival, and adhesion [223]. As shown by recent studies, CD155 was considerably higher in patients with AML in comparison with the control cases, and it appears that there is an association with a poor prognosis [224]. In spite of CD155's normal expression on hematopoietic cells, it has an immunosuppressive role and a dual function in tumor immunity. In order to maintain normal NK and T-cell function, CD155/CD226 (stimulatory molecule) must be in balance with CD155/TIGIT or CD155/CD96 (inhibitory molecules). Nevertheless, in TME, this balance may be disrupted by inhibitory signals mediated by decreased CD226 and increased TIGIT. Additionally, during a study on osteosarcoma, western blot analysis demonstrated that the CD155 blockade reduces metastasis through the downregulation of phosphorylated FAK (pFAK) and focal adhesion kinase (FAK) [225]. Taken together, there is an association between overexpression of CD155 and tumor progress and a poor prognosis in tumor cells, and it may become a potential targeted therapy for AML patients [226]. Poliovirus-Rhinovirus Chimera (PVSRIPO) is an oncolytic viral therapy that promotes tumor cell death by binding specifically to CD155, causing the tumor cell lysis and the release of Danger-Associated Molecular Patterns (DAMPs), which result in T and NK cells becoming more activated and inhibiting tumor growth. Besides, it has been shown that CD155-expressing dendritic cells and macrophages involve T cells and confine viral replication with exposure to PVSRIPO [227–229]. Therefore, using PVSRIPO could be a potential therapeutic approach for AML and researchers can consider it in future investigations to target CD155.

Additionally, OMP-313M32, BMS-986207, MTIG7192A, and MTIG7192A are anti-TIGIT antibodies that have been clinically tested alone or combined with nivolumab and atezolizumab [230].

#### CD200

CD200, as CD200 receptor (CD200R) ligand, is an immunosuppressive receptor that expresses on myeloid and lymphoid cells and is thought to be an inhibitory IC [231]. Previously, Herbrich et al. developed a novel bioinformatics approach that analyzed widely available AML gene expression datasets and found CD200 as substantially over-expressed in AML stem cells (LSCs) when compared with paired blast cells, in addition to their normal hematopoietic stem cell counterparts [26]. Specifically, CD200 appears to have an immunosuppressive impact on NK cells and macrophages, and correlates with a high frequency of FOXP3^+^ Treg cells, as well as being identified as a poor prognostic factor in AML [26]. TTI-CD200 is an anti-CD200 antibody that was investigated by Diamanti et al. and Rastogi et al. on leukemia-propagating cells (LPCs) and mice model respectively. Both in vitro and in vivo experiments indicated that the anti-CD200 antibody significantly reduced disease burden and extended the survival of the cells and mice [232, 233]. Studies suggest CD200 is a hallmark of metastasis in cancers such as CML, breast, lung, bladder, prostate, melanoma, and squamous cell carcinoma [234, 235]. Although the role of CD200 in AML metastasis is not well understood, future studies could be valuable for evaluating its potential role in AML metastasis.

## The role of metabolic processes of BMM cells in AML angiogenesis, metastasis, and cell proliferation

**Fig. 2:**
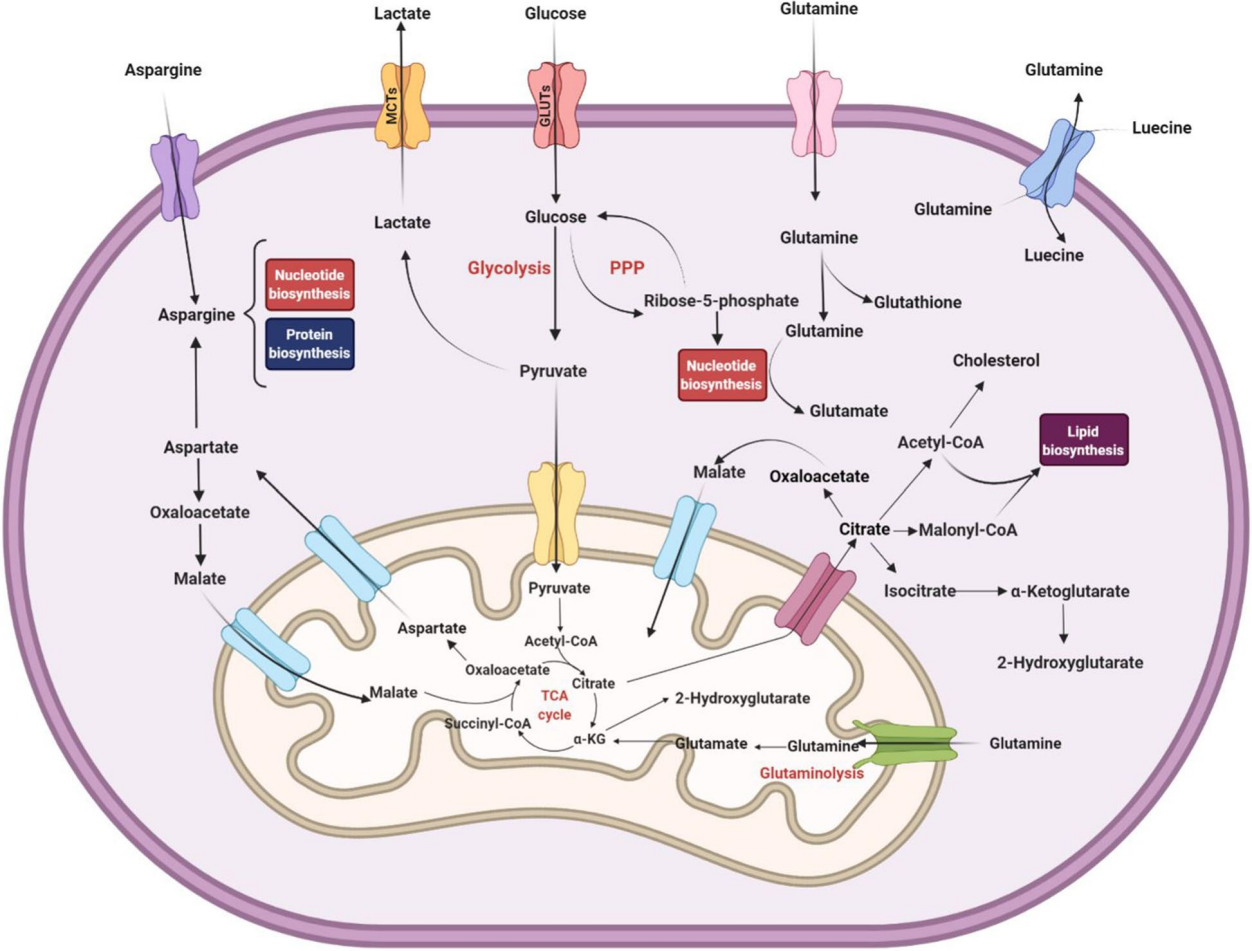
The metabolism of an acute myeloid leukemia cell. Metabolic reprogramming produces ATP and intermediates for the biosynthesis of amino acids, nucleotides, lipids, and redox components which required for high proliferation rate. Flexible changes in nourishing and processing BMM and leukemic cells in ecological conditions propel significant differences in the AML BMM cells resulting in these substances happening in the preexisting metabolic pathways to AML advancement. GLUTs: glucose transporters; MCTs: monocarboxylate transporters; α-KG: α-Ketoglutarate; PPP: pentose phosphate pathway

AML is one of the most lethal and frequent leukemias. The aggressiveness of the disease, which is still resistant to treatment, is related to its broadly diverse and dynamic metabolism. AML cells have metabolic plasticity and dominate normal hematopoietic cells by utilizing multiple nutrient sources for biomass and energy supply. In this section, metabolic checkpoints in AML are discussed (Fig. 2). We suggest that future studies should thoroughly explain the importance of these metabolic checkpoints on leukemic cells. Furthermore, these checkpoints should be regarded as potential multi-targeted treatments for AML when used alongside other conventional therapies.

### Glycolytic metabolism reprogramming in AML

The special requirement of cancer cells on glucose absorption and consumption is entirely acknowledged. Cancer cells, as reported by Otto Warburg in 1924 [236], do not fully use mitochondrial metabolism to utilize glucose-derived pyruvate. Instead, cancer cells transform pyruvate to lactate, due to low ATP yields. The Warburg effect, as a crucial anabolic mechanism, enables cancer cells mastering cell proliferationand growth and is affected by oncogenes that intercept growth factor signaling pathways [237]. Different shunts from glycolysis, mostly the pentose-phosphate pathway (PPP), allow cancer cells to obtain the nucleotides, electron carriers, and amino acids required for tumor growth. As a result, cancer cells have a great dependency on glycolysis not so much for energy generation as for the synthesis of building blocks [238]. The Warburg effect provides a good equilibrium between the anabolic roles of glycolysis and energy resources, as well as their variations, by ensuring a steady action of glycolysis (Fig. 2).

#### AML cells high dependence on glucose utilization

Some researchers indicate that AML cells consume a lot of glucose. Cunningham et al. in 2016, using 18F-Fluoro-deoxy-Glucose (18FDG) as a marker in individuals with AML (*n* = 124) showed that glucose absorption in AML bone marrow was uniformly high [239]. In a small group of AML patients, Herst et al. have found that an increase in aerobic glycolysis upon diagnosis is indicative of better treatment efficacy and lifespan [240]. Chen et al. have analyzed serum samples from patients with AML (*n* = 400) with 446 normal participants and discovered that serum from patients with AML has a unique glucose metabolic profile, with major changes in six metabolites in this pathway [241]. Based on their study, Pyruvate, Lactate, 2-HG, 2-oxoglutarate, and glycerol-3-phosphate were all related to a decreased survival rate. There were no major differences between different WHO AML subtypes, indicating this metabolic profile as representative of a persistent component of AML regardless of cytogenetic risk groups [241]. In relation to the low-glycolytic cell line HL-60, studies on four AML cell lines (U937, THP-1, KG-1, and OCI-AML3) indicated the increase in the expression of varied TCA and glycolytic genes. Proliferation was reduced when glycolytic inhibitors were added to the mix. Additionally, knocking down hexokinase-1 (HK-1) in OCI-AML3 cells and U937, and treating the aforementioned AML cell lines and actual AML blasts with the glycolysis blocker 2-deoxy-D-glucose (2-DG), enhanced the chemotherapeutic drug cytosine arabinoside (Ara-C) sensitivity [241].

### Metabolism of cytosolic carbohydrates

The glucose transporters are essential for cellular glucose absorption (GLUTs). GLUT family members with high expression profiles have been found in a variety of tumors [242–244]. Several studies focused on the expression of GLUTs in AML cells, the processes by which they are regulated, and the relationship between GLUT expression and patient treatment effects. Enhanced GLUT1 mRNA expression is related to low chemotherapy response in one patients group [245, 246]. Compared with the cases in complete remission and controls, Sun et al. have found that the long noncoding RNA (lncRNA) antisense RNA at the INK4 locus (ANRIL) is increased in patients with AML of various phases. In vitro, knocking down ANRIL enhanced senescence in MOLM-13 and HL-60 cells. A system with adiponectin receptor 1 (AdipoRl), the cell energy sensor adenosine monophosphate -kinase ex. (AMPKcx.) and sirtuin-I (SIRTl) effectively modulate GLUT1 protein expression and stimulate glucose metabolism [247].

The FLT3-ITD (Ba/F3/ITD) upregulation in the murine lymphoid cell line Ba/F3 resulted in a high reliance on glycolysis, with sensitivity to pharmacologic impedance. Furthermore, when the FLT3 inhibitor sorafenib was combined with glycolytic inhibitors, it caused a dramatic increase in the FLT3-inhibitor sorafenib cytotoxicity. It implies the potential adaptation of cells to FLT3-ITD-driven glycolysis and be particularly sensitive as a result [248]. A recent discovery of the lncRNA urothelial carcinoma-associated 1 (UCA1) points to hexokinase-2 (HK-2) as a potentially crucial molecule linking AML oncogenic action with glycolytic adaptation. HK2 inhibitors are considered pyruvate analogs, such as 3-bromopyruvate and benitrobenrazide, which are highly reactive and are perceived to be HK2 analogs [249].

UCA1 has been implicated in the oncogenic function of CCAAT/enhancer-binding protein-ex dominant-negative isoform (C/EBPcx-p30)-positive AMLs [250] and in the chemoresistance of AML cells to daunorubicin-based therapeutics [251]. UCA1 also operates as a competitive endogenous RNA (ceRNA) of miR-125a, inhibiting its suppressive effect on HK-2 gene expression [252]. Pathways dependent on PI3K/protein kinase B (PKB/AKT) activate HK enzymes, which induces the first stage of the glycolysis pathway. The HK-2 is a substance of chaperone-mediated autophagy (CMA) in AML cells. By interacting with the chaperone Hsc70, specific proteins with a CMA-targeting motif are transported to the lysosome, interacting with the lysosome-associated membrane protein type 2A (LAMP-2A) in CMA [253]. Xia et al. emphasized the dependence of HK-2 on CMA and demonstrated that inhibiting autophagy and FLT3 simultaneously activates CMA, resulting in cancer cell death under normal nutritional occasions [254]. For cell growth, a benefit of highly active glycolysis is the passage of glycolysis intermediates through other pathways to produce biosynthetic building blocks. The glucose-6-phosphate (G6P) product of HK-2 is an entry point into multiple different processes, such as the PPP, glycogenesis, and hexosamine synthesis process. The PPP is an important pro-survival pathway in AML and its action is due to the role of the central cell signaling cascade mammalian target of rapamycin complex 1 (mTORC1) [255].

Poulain et al. have discovered that 6-aminonicotinamide, a significant inhibitor of G6P dehydrogenase (G6PD), causes apoptosis in primary AML blasts and AML cell lines but not in standard hematopoietic progenitor cells. Poulain et al. showed that inhibiting mTOR causes metabolic reprogramming [256]. Additionally, they found a considerable elevation in the oxidative metabolism of TCA cycle and glycolysis inhibitor resistance when they inhibited both mTOR and G6P at the same time [255]. These findings are consistent with those of Chen et al., which found a decrease in the PPP intermediary D-ribose phosphate in serum samples of AML, implying greater PPP and purine synthesis activity [241]. The relevance of the PPP in AML is maintained by the elevation of PPP genes in 61 percent of cases with AML [257].

These findings suggest that the PPP, particularly G6PD, may be used to establish effectively targetable metabolic requirements for AML. AML hematopoiesis is organized, similar to normal bone marrow, with just a trivial portion of AML cells, called LSCs, important for disease persistence and relapse upon effective therapy. Expression investigations of rate-limiting components in metabolic pathways in most of AML cultures are thus of limited relevance for our knowledge of critical AML strategies unless they are backed up by more definite functional data. In an MLL-AF9-based in vivo AML model, In the study by Saito et al. the AMPK depletion effect was investigated [258]. They discovered that knocking down AMPK resulted in the LSCs loss and a longer delay of AML formation after transplantation. AMPK deletion in vitro resulted in the GLUTs removal from the leukemic progenitor cell membrane, as well as decreased accurate glucose uptake, the production of ROS, and cell death, especially when glucose levels were low. Chen et al. (2016) described an insightful and potentially therapeutic targetable version of the approach [259]. They discovered that AML cells might adapt to low glucose levels by the upregulation of SLC2A5, the fructose transporter GLUT5 gene. Fructose can be easily transported to glycolysis by fructose kinase. Patients with AML who might have high levels of GLUT5 get a worse prognosis compared to those with low levels of GLUT5. In vitro, inhibiting fructose absorption with drugs increased Ara-C cytotoxicity. The anti-cancer properties of traditional Chinese compounds have been investigated in detail and include Oleanolic acid, Carpesium abrotanoides L, Tanshinone IIA, Dioscin, Polyphyllin VI, Euxanthone, Chrysin, etc. many of these compounds affect glucose enzymes and/or transporters, including GLUT1, HK2, LDHA, HIF-1α, PFK1, and PKM2 [260].

A recent study highlighted the value of appropriate glucose concentrations in AML. Leukemia cells cause complicated manipulations of the host's metabolic homeostasis. Ye et al. 2018 discovered that when compared to normal hematopoietic cells, AML cells use up to 20 times more the amount of glucose. The researchers showed that leukemia cells control different host organs in order to maintain high glucose levels in the bone marrow. So, increased peripheral insulin tolerance and insulin secretion suppression are two of the identified outcomes. In respective mouse models, a therapeutic approach to treat the insulin resistance phenotype increased survival and reduced leukemia development [261].

### Mitochondrial metabolic pathway in AML Biology

#### Hematopoietic and leukemic stem cells switch between oxidative phosphorylation (OXPHOS) and aerobic glycolysis

The liver-type (PKL), red blood cell isoform (PKR), and muscle isozyme M1 and M2 (PKM1 and PKM2) are the four-pyruvate kinase (PK) variants found in mammals [48]. PKM2 is nearly often the dominant PK isoform in cancer cells, and this isoform has been demonstrated to significantly promote pyruvate departure from the TCA cycle and OXPHOS in a landmark publication [262]. The procedure is still unclear. PKM2, on the other hand, has been demonstrated to have a variety of roles, and its function as a switcher between OXPHOS and aerobic glycolysis could be independent of its function as a rate-limiting glycolytic enzyme [263].

By using natural products as the starting material, Li J et al. showed that micheliolide (MCL) selectively activated PKM2 via covalent binding at cysteine424 (C424), which does not occur in PKM1. As a result of this interaction, the formation of tetramers is increased, K433 is inhibited from being acetylated, and PKM2 can be translocated into the nucleus [264].

The lactate dehydrogenase (LDH), which catalyzes pyruvate to lactate, is another effective inhibitor of aerobic glycolysis. For AML patients, high serum LDH level upon diagnosis is an important negative prognostic indicator [265, 266]. The significance of the shift between OXPHOS and aerobic glycolysis for LSC functioning and AML therapeutic efficacy is a major unresolved issue in AML studies. The function of normal HSCs is significantly affected by this change, according to valuable research. Pyruvate is directed into the mitochondria for the TCA cycle, and its dehydrogenation to acetyl-CoA occurs in this phase as a primary source for the TCA cycle. Several isoforms of pyruvate dehydrogenase (PDH) kinase (PDK) phosphorylate and inactivate (PDH), the enzyme catalyzing this critical phase at the start of TCA cycle. Takubo et al. utilized single-cell metabolome analysis to show that Pdk4 and Pdk2 loss leads to losing HSC self-renewal potential [267]. Yu et al. reported the alternative in the same issue of Stem Cells. They discovered that specific deletion of the mitochondrial phosphatase protein tyrosine phosphatase mitochondrial 1 (PTPMT1) gene led to fast hematopoiesis failure. PTPMT1 is required for the transport of pyruvate into the mitochondria, where it is employed for the TCA cycle as a fuel [268].

Thus, in regular HSCs, the shift from aerobic glycolysis to OXPHOS is both adequate and essential for the recruitment of inactive HSCs into hematopoiesis and the fast elimination of self-renewal capability of this population. Previously, metabolic changes in HSCs were considered a reaction to changing needs of cell fate choices mediated by epigenetic, transcriptional, and signaling pathways. It appears that the metabolic mechanisms control the destiny of HSCs in this case. How metabolic processes influence cell destiny choices is still unknown [269]. Researchers discovered that depleting PKM2 and LDHA at the same time, a state that favors pyruvate metabolism in mitochondria, significantly reduced leukemia onset and survival. In this scenario, the double deletion had a minor impact on normal hematopoiesis particularly in the lack of HSC stress. In general, available evidence suggests that AML is strongly dependent on high glycolysis levels. As a result, a medical approach that causes a shift from aerobic glycolysis to mitochondrial respiration could inhibit AML blasts from proliferating. However, it seems that the OXPHOS function and TCA cycle is a critical prerequisite of AML and is described further in the following.

#### AML cells krebs cycle and OXPHOS

Mitochondria are the vital point in the cells, in which carbohydrates, fatty acids, and amino acids are metabolized and enter into the Krebs cycle and an electron transport chain (ETC) that produces energy via oxidative phosphorylation. The relevance of TCA cycle as a possible beneficial target was investigated in clinical experiments using the compound CPl-613. PDH (which catalyzes the pyruvate to acetyl-CoA) and α-KG dehydrogenase (that converts α -KG to succinyl-CoA) are both inhibited by CPl-613. CPI-613 decreases the levels of oxygen in AML cells [270]. Farge et al. used Ara-C for treating AML patient-derived xenograft-carrying mice and investigated the AML metabolism function in resistance to chemotherapy [271]. They discovered that the cellular increase of mitochondrial metabolism is a key feature of Ara-C resistance in AML. Based on their observations, AML cells show greater mitochondrial densities and higher OXPHOS levels ex-vivo. OXPHOS inhibition made the cells more sensitive to Ara-C and shifted resistance. An increase in mitochondrial function genes was found to be inversely associated with Ara-C sensitivity, and there was an association with a poor prognosis in a current transcriptome analysis of AML [272]. In another research, increased OXPHOS (mediated by MYC and MCU) in breast cancer stem cells resulted in the accumulation of HIF-1α and the development of chemoresistance [273]. Enhanced ROS levels, which were caused by active OXPHOS, induced HIF-1α accumulation. HIF-1α inhibition decreased the tumorigenic potential of these cells and altered resistance. Though other researchers propose that OXPHOS can significantly contribute to chemoresistance, additional studies are required for further knowledge of the molecular mechanisms.

Finally, researchers have identified mitochondrial metabolism as a significant response factor for venetoclax, a significant BCL-2 protein–protein interaction inhibitor [274, 275]. Venetoclax is approved for treating various types of lymphoid cancers. Its therapeutic efficacy in AML has been proven [276], particularly when used in conjunction with hypomethylating compounds and reduced Ara-C [277, 278]. Complete response rates in previously untreated old individuals reached up to 67 percent [278] that has the potential to profoundly alter how AML is treated in the future. The FDA approved venetoclax combined with hypomethylating compounds for patients with AML. The Jordan group compared the metabolic activity of AML LSCs to non-LSCs and discovered their selective dependence on OXPHOS energy sources. Besides, these authors discovered the suppression of OXPHOS by venetoclax in these cells and the link between these metabolic changes and venetoclax toxicity [274]. A current metabolic loss-of-function analysis in AML cell lines produced similar results. The research also indicates the deletion of some Krebs cycle components, including the succinate dehydrogenase complex subunits SDHC and SDHA, and proteins that facilitate the Krebs cycle to be using glutamine as a fuel sensitizes AML cells to venetoclax treatment [275]. Combining venetoclax and the hypomethylating compounds azacitidine or decitabine demonstrated hopeful synergistic consequences in a recent phase 1b clinical investigation [277, 278]. The Jordan team also observed that venetoclax's efficacy was associated with selective targeting of the LSCs capability of patients for performing oxidative phosphorylation [279]. According to another research, a potential mechanism could be caused the reduced availability of amino acid as a fuel for the Krebs cycle that is solely dependent on LSCs from AML patients that are newly diagnosed [280]. It seems that LSCs from AML recurrence cases who are more likely to develop resistance to venetoclax therapy increase FAO pathways, implying that they may develop resistance to the combination by acquiring extra metabolic flexibility.

#### AML cells mitochondrial capacity and respiratory function maintenance

Electron passage from metabolic intermediates to the final oxygen acceptor is one of the most essential mitochondrial processes. Mitochondria generate an electrochemical gradient in the inner mitochondrial membrane to do this. The formation of ROS comes from a mismatch between the ETC's activity and metabolic requirements. Because AML cells have an elevated mitochondrial mass and a smaller reserve respiratory capacity per mitochondrion, they seem to be more susceptible to oxidative damage than other hematopoietic cells [281]. In a chemical screening, tigecycline was found to exclusively suppress the AML cell proliferation but not the proliferation of normal hematopoietic progenitors, validating this concept [282]. This chemical is a powerful inhibitor of mitochondrial translation, causing respiratory chain components to be reduced even further. They showed evidence that the drug's anti-leukemic properties were related to this feature. In a phase I trial, tigecycline was shown to be safe when used as a single drug in patients with recurring AML. Moreover, the drug's pharmacokinetic characteristics were unexpectedly unfavorable, and there were no indicators of antileukemic effect in vivo [283]. Finally, the researchers looked for another way to target the higher mitochondrial load in AML as targeted therapy. As discovered by these authors, 2′3'dideoxycytidine, as a reverse transcriptase inhibitor for HIV treatment, is especially harmful to AML cells since it efficiently limits mitochondrial DNA replication [284]. Cole et al. (2015) found that the mitochondrial ATP-dependent Clp protease proteolytic unit (ClpP), interacting with respiratory chain proteins, is considerably increased in a considerable number of patients with AML [285]. ClpP deletion is lethal for ClpP-expressing leukemic cell lines, e.g., the AML cell line OCI-AML2. ClpP deficiency prevented mitochondrial energy generation in the cells. ClpP knockout mice were alive without a phenotype in hematopoiesis in the same study. ClpP is thus not required for the usual HSC function. Treating with A2-32-0l as a ClpP inhibitor decreased the transplanted OCI-AML2 cell line growth in a xenograft experiment (SCID animals). ACS-010759 and ME-344 also inhibit oxidative phosphorylation (OXPHOS) by targeting the electron transport chain (ETC). Imprisoned family of mitochondrial protease inhibitors (ONC201, ONC206, ONC212) act by activating ClpP mitochondrial protease and reducing the activity of certain important pathways [286].

Mitophagy is a type of selective autophagy with dependence on the autophagy receptor p62 expression, which is another method of mitochondrial quality control. It has been shown that cells manage the number of dysfunctional mitochondria and their position through this process. In AML mouse models, loss of p62 prevents mitochondrial clearance from AML blasts, prolonging disease latency [287]. The active transmission of entire mitochondria to AML blasts from bone marrow stromal cells is another controlled process in AML [288, 289]. It allows for the proper amount and function of mitochondria to be maintained. It was found that enzyme NADPH oxidase-2 (NOX2) catalyzes this process, which is required for creating tunneling nanotubes by for leukemic cells and has also been identified as a protective response to oxidative stress in normal hematopoiesis [289]. The decrease of NOX2 in AML cells led to a reduction in respiration capacities, implying the importance of this energy transfer pathway in AML blasts. Compared with the controls with NOX2-replete AML line transplantation, transplanting a NOX2 knockdown human AML cell line into NSG mice increased survivability. To conclude, AML cells are on the verge of losing their respiratory system due to increasing mitochondrial mass, decreased ETC capability, and constant oxidative stress. It seems that there in AML, which is highly different from normal hematopoiesis, there is a sensitive balance between the prerequisite for ROS-induced stress, ETC reserve, OXPHOS-dependent energy supply, and other mitochondrial roles, which are critical for cell survival, proliferation, and fate. Studying how this ratio is regulated could lead to the discovery of AML-specific deficiencies.

### The Metabolism of Amino Acids in AML

Amino acids are essential elements for protein expression and intermediate compounds in biosynthetic pathways, making them crucial in organisms. Researchers have identified various amino acids for filling up, generating, or supplying into anabolic and catabolic pathways in cancer cells to a variable level than in normal tissues [290–292]. Most information in AML refers to changes in glutamine, BCAA, and arginine metabolism.

#### Glutamine’s role in AML cells

Glutamine acts as a coordinator for a wide range of cell processes. Glutamine can be synthesized de novo in the cell, imported by the glutamine importer SLC1A5, generally referred to as ASCT2, or synthesized by the lysosomal breakdown of proteins collected through endocytosis, autophagy, or macropinocytosis. Its essential function in metabolism is related to the fact that it is possible to convert glutamine into ex-KG through a variety of metabolic pathways.

Glutamine is transformed into glutamate in the first stage by the enzyme glutaminase (GLS). It is possible to directly convert Glutamate into α -KG by glutamate dehydrogenase (GLUD), or deaminate and used as a nitrogen source in producing non-essential amino acids and pyrimidine and purine nucleotides in a variety of processes [293]. As a metabolite of the Krebs cycle, it is possible to supply α-KG into the Krebs cycle in a process called anaplerosis, which is commonly used by cancer cells. Meanwhile, the cell can use α-KG as a carbon donor in FAS, in reducing NADP^+^ to NADPH that is required as an electron donor and in glutathione production, one of the most important ROS scavengers, which is particularly important in cancer cells because of the enhanced ROS generation. Lastly, intracellular glutamine levels have a crucial function in controlling signaling pathways, especially mTORCl activity.

Although leucine, rather than glutamine, controls the key switch between cellular ana- and catabolism, glutamine plays a critical regulatory role because the amino acid transporter LAT1 needs the release of one molecule of glutamine to import one molecule of leucine [294].

It has been shown that Gln antagonists, like DON (6-diazo-5-oxo-l-norleucine) impedes enzymes in enzyme GLS inhibitors or Gln utilization, BPTES ((bis-2-(5-phenylacetamido-1,2,4-thiadiazol-2-yl) ethyl sulfide, compound 968 (5-(3-bromo-4-(dimethylamino) phenyl)-2,2-dimethyl-2,3,5,6-tetrahydrobenzo[a]phenanthridin-4(1H)-one) and CB-839 are beneficial in several cancer types in vivo, in vitro and in different clinical trials ((NCT04250545, NCT03965845 and NCT03904902) [295, 296].

#### AML cells requirement on arginine

The amino acid arginine is essential for cells for a variety of biological processes. The amino acid is a key component of protein synthesis, needed to produce a variety of metabolites, such as polyamines and nitric acid. Different types of cancer have demonstrated higher arginine requirements within tumor development, and it has been proposed that preventing arginine metabolism can be used as a possible cancer medical strategy [297].

Argininosuccinate lyase (ASL) and argininosuccinate synthetase-1 (ASS1) hydrolyze citrulline to produce arginine. Because a significant number of AMLs lose ASS1, they must need an exogenous arginine supply obtained through the diet [298, 299]. The arginine transporters CAT-2B and CAT-1 are expressed by AML cells in a consistent way, and plasma arginine contents in patients with AML are much lower than in normal individuals [299]. Exogenous arginine limitation led to a decrease in cell survival. The effects of the arginine deiminases ADI-PEG 20 and BCT-100 on AML cells were investigated in vivo and in vitro. BCT-100, as a recombinant arginase, reduces arginine both intracellularly and extracellularly. The drug repressed the growth of HL-60 xenografts and decreased the proliferation of new AML blasts in vitro [299]. This impact was increased when Ara-C was added to the mix [299]. In sensitive basic AMLs and xenograft models, ADI-PEG 20, a mycoplasma-derived arginine deiminase, decreased AML risk. In cells with resistance to ADI-PEG 20 as a single agent for argininosuccinate synthase 1 (ASSl) overexpression, ADI-PEG 20 increased Ara-C sensitivity with stimulatory activity in cells with resistance to ADI-PEG 20 also as a topical treatment [298]. Overall, the research cited above revealed that dependence on arginine is a metabolic feature of AML exacerbated by the absence of ASSl. ASSl is the enzyme allowing the production of arginine de novo. Promising results have been obtained in initial clinical trials with ADI-PEG 20 in lung cancer, AML, and ASSl-deficient malignant pleural mesothelioma [300–302]. AML patients are being treated in a clinical trial with BCT-100 (https://clinicaltrials.gov/ct2/show/NCT03455140).

#### The branched-chain amino acid in AML

BCAAs include valine, isoleucine, and leucine. The BCAA transaminases 1 (BCAT1) and 2 (BCAT2) catalyze their production in a reversible transamination process. BCAAs have been demonstrated to be critical nutrients in the treatment of several tumors. In different types of cancer, particularly breast and liver cancer, it has been found that there is a link between the BCAT1 overexpression and an invasive tumor progression and appearance [303]. AML physiology has recently been found to be influenced by changed BCAA metabolism and increased BCAT1 levels. The Trumpp lab [304] demonstrated the significant overexpression of BCAT1 in AML LSCs using quantitative expression proteomics on organized populations of primary AML bone marrow samples. This lab also discovered that overexpression of BCAT1 led to increasing α-KG amination and, as a result, lower intracellular amounts of α-KG. Alterations in epigenetically active α-KG-dependent dioxygenases, such as the TET and EGLN1 family enzymes, resulted in BCAT overexpressing LSCs having identical epigenetic alter as IDH-mutant LSCs. Moreover, there was a link between the overexpression of BCAT1 and a poor prognosis in cases without IDH mutations, but only to a reduced amount in patients with IDH mutations.

Hattori et al. demonstrated that BCAT1 is increased in chronic phase CML to blast crisis and it is significant in de novo AML. BCAAs, which are the metabolites of BCAT1, were found to be the culprit for the impact on malignancy progression in metabolomic studies. Gabapentin, a BCAT1 inhibitor, substantially inhibited the clonal expansion of actual AML cells and AML cell lines [305]. Because BCAT1's effects appear to be LSC-specific and gabapentin is commonly accessible and suitable for clinical application, it is fascinating to observe if clinical ways to utilize BCAT1 as a targeted therapy in myeloid leukemias are being developed.

### Lipid function in AML

As noted above, AML relies on biomass and energy generation to meet the demanding requirements of cell growth and proliferation. Lipids are primarily used in mammals as a source of NAD(P)H, ATP, and constituents for specialized lipids that as crucial signaling components. FAs are delivered to cells through transporter-mediated FA absorption, triglyceride hydrolysis, or de novo FAS.

FA absorption is accomplished through mitochondrial β-oxidation, commonly called FAO, which produces flavin adenine dinucleotide (FADH2), NADH, and acetyl-CoA, and all of them contribute to the TCA cycle and OXPHOS metabolic processes. However, after being converted to citrate in the TCA cycle, acetyl-CoA from FAO can be transported to the cytosol and then used to synthesize NADPH [306]. FA absorption and utilization have been demonstrated to mediate essential factors of AML biology, such as LSC fate choices, adjustment to a unique microenvironment, and therapeutic response/resistance recently. The Andreef lab provided the first evidence that FA metabolism could have a function in AML, showing that downregulation of CPT1 (suppression FA translocation to mitochondria) increased sensitivity of AML to apoptosis-inducing agents interfering with the mitochondrial apoptosis elements. According to these findings, it could be related to an FAO function that has nothing to do with ATP generation. FAO controls the BAK-dependent mitochondrial permeability transition, which is a crucial element of cytochrome c-dependent apoptosis regulation [307]. A large number of studies suggest that AML cells, particularly LSCs, are necessary for high rates of FAO and low FAS action. In AML, the α-KG-dependent dioxygenase PHD3 is down-regulated that regulates the rate-limiting FAS enzyme, switching it on and FAO) [308]. As a CPT1 inhibitor, Etomoxir has been reported in several studies to be able to resensitize resistant LSCs to venetoclax with azacitidine (ven/aza) therapy [309–311].

Despite supplying AML cells with sufficient fuel for FAO, these cells are unnecessary to turn on FAS. For instance, CD36-positive LSCs exist in a specific niche in gonadal adipocytes in a mouse CML blast crisis type, where they are preserved from cytotoxic therapy and LSC depletion by FAO [312]. In patients, the presence of a CD36-positive LSC fraction with distinct metabolic properties was validated. In an investigation of the chemotherapy resistance process in primary AML cells, Ara-C was used to treat mice with patient-derived xenografts from AML cases, and the metabolic patterns of the resistant cells were examined. Also, the resistant AML cells had considerable membrane overexpression of the FA transporter, CD36, and elevated FAO levels, in order to have success predominance of OXPHOS and high levels of ROS [271]. In the case of venetoclax resistance, a similar pattern was found about FAO. Venetoclax response is increased in AML cells with high OXPHOS-fueled by amino acids, as mentioned in this review. The increased FAO in the returned cases was a notable difference between venetoclax-resistant reverted AML cases and venetoclax-responsive de novo disease. As a result, FAO could play a role in a greater mechanism of therapeutic resistance. It should be noted that lipid deprivation did not affect the survival or colony-forming capacity of LSCs in total [280]. In studies, AML cells were supplied with bone marrow adipocytes, which activated AMPK in the cells and induced a transcriptional pathway that increases growth and FAO [97]. Reducing intracellular free fatty acid trafficking into AML cells, led to AML mice models living much longer [95]. Therefore, FA feeding of AML cells seems to be a necessary condition for leukemic growth, apoptosis induction, and cytotoxicity resistance. Essential points of this metabolic circuitry include cellular uptake of FAs through CD36 and FA scrambling into mitochondria with the CPT1 support. FAO supplies into the Krebs cycle and ETC pathways, which appears to be a key strategy for supporting self-renewal in both normal HSCs and LSCs—when the major result of glycolysis, pyruvate, does not power mitochondrial metabolism. Moreover, it is unclear which factors downstream of FAO are important for leukemic development. One reason is that FAO is a major source of NADPH, as it generates citrate and acetyl-CoA in mitochondria, which is then used by the enzymatic processes in the cytosol to make NADPH [306]. Jeon et al. discussed the FAO significance for NADPH homeostasis in cancer cells. NADPH production by the PPP is reduced under metabolic stress, and higher AMPK levels enhance FAO-derived NADPH synthesis by reducing NADPH-consuming FAS [313]. NADPH generated by FAO could be the important cellular electron donor for AML cells undergoing treatment to resist oxidative stress and participate in anabolic activities required for cell proliferation. An important study on Avocatin B provides evidence that this is the case. Lee et al. discovered Avocatin B as a strong anti-leukemic drug in vitro after evaluating a natural substance library on specific leukemic cell toxicity. Avocatin B is a lipid generated from avocado fruit with an unusual number of carbon atoms. It was discovered that inhibiting FAO produces a 50% drop in NADPH levels, and then ROS-mediated apoptosis happens. In leukemic cells lacking CPT1, Avocatin B activity was reduced [314]. With co-culturing AML cells with bone marrow adipocytes and feeing with Avocatin B [315], the cells adjusted by enhancing glucose and FA absorption, likely due to AMPK and activation of activating transcription factor 4 (ATF4). ROS levels rose, making the cells highly vulnerable to Ara-C chemotherapy.

## Signaling pathways stimulated in AML cells by BMM factors that lead to AML progression

**Fig. 3:**
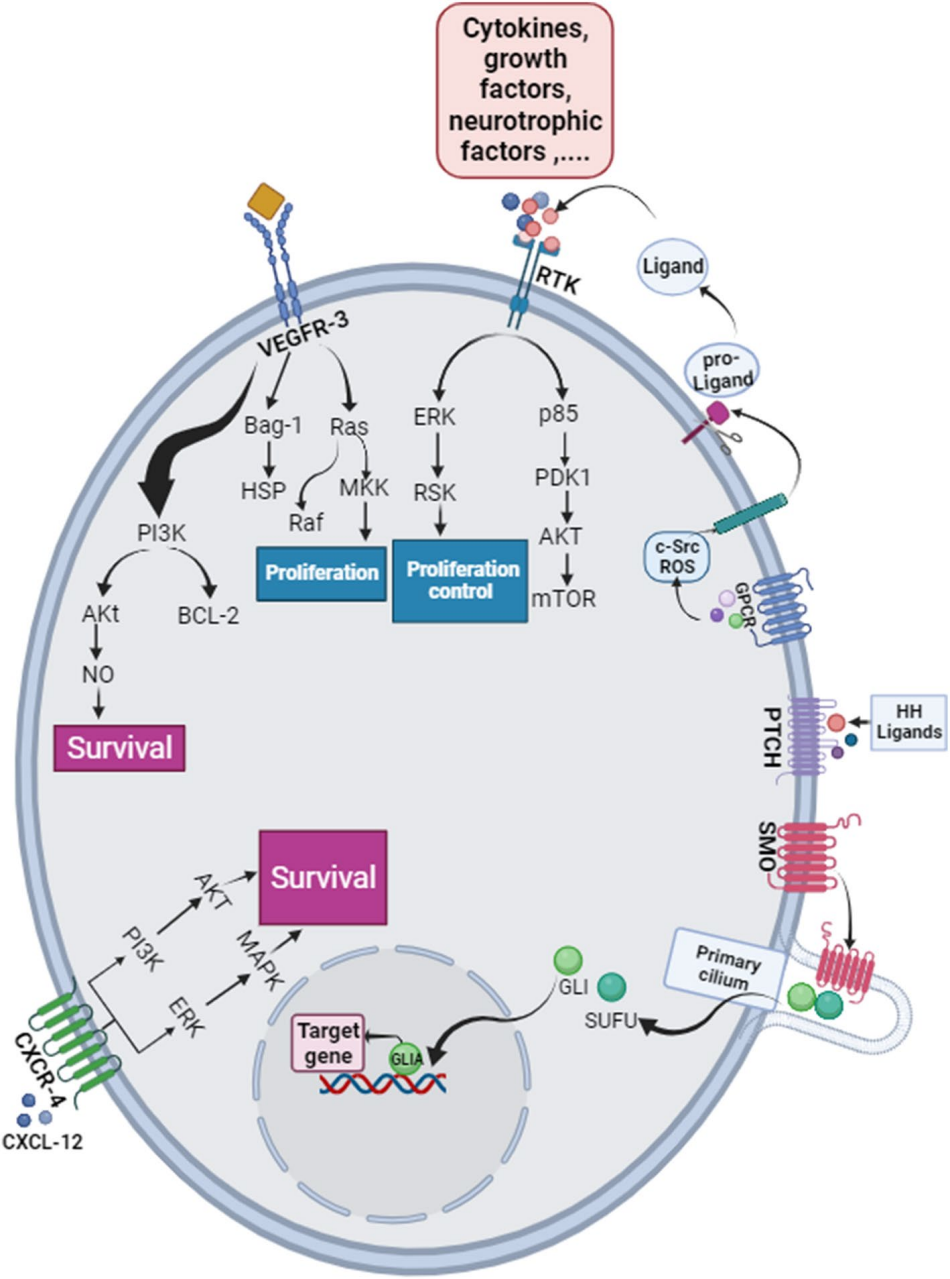
The illustration indicates several pathways in AML cell: 1. CXCL-12/CXCR-4 axis initiates PI3K and ERK signaling pathways that leads to survival of AML cells through AKT and MAPK respectively. 2. Interaction of VEGFR-3 with its ligand (VEGF) starts PI3K/AKT/NO (survival), PI3K/BCL-2, Bag-1/Hsp, Ras/Raf (proliferation) and Ras/MKK (proliferation). 3. Several RTK ligands such as cytokines, growth factors, neurotropic factors, etc. interact with RTK that results in ERK/Ras (control of proliferation) ans p85/PDK1/AKT/mTOR. 4. some intracellular factors such as ROS and C-src are activated by GPCR. In the next step infiltration of these factors to cell membrane surface causes cleavage of proligands to ligand form of RTK. 5. HH ligands intract with PTCH. This interaction leads to inhibition of inhibitory activity of PTCH. When PTCH is inhibited, SMO can move toward the primary cillium and cause separation of GLI from SUFU. GLI in the nucleus promotes transcription of some target genes

The BMM contains a variety of cells whose interaction with AML cells triggers multiple signaling pathways (Figs. 2 and 3). Interestingly, AML cells spontaneously produce VEGF molecules that affect intra/extracellular VEGF receptors (VEGFR) that are expressed on AML cells [316]. VEGFA and VEGFC are frequently expressed in AML cells [90]. VEGFR-3 is highly expressed on the cytoplasmic endosome while VEGFR-2 is transferred from the cytoplasmic membrane to the cytoplasmic endosome and nuclear membrane upon activation [317]. Importantly, VEGF blockade via anti-VEGF antibodies prevents VEGFR-2 translocation to the nuclear membrane and it is clear that the transfer of VEGFR-2 is largely dependent on its binding to VEGF [318].

The development of anti-VEGF or anti-VEGFR therapies for several cancer types such as AML has resulted in some approvals. As an example, Vorolanib (CM082) is a minimally accumulating multi-targeted tyrosine kinase receptor inhibitor with a short half-life. By inhibiting the proliferation of human umbilical vein endothelial cells (HUVECs) and the formation of HUVEC tubes in vitro, vorolanib inhibited the proliferation of VEGF-induced cells [319].

The anti-tumor effect of vorolanib was dose-dependent in mouse xenograft models of MV-4–11, A549, 786-O, HT-29, BxPC-3, and A375 cells. On MV-4–11 xenografts, complete tumor regression was also achieved without significant toxicities, however, significantly lower body weights were observed when Vorolanib was administered at 40 mg/kg qd [319].

A study was also conducted to test curcumin's ability to inhibit VEGF in leukemic cell lines (U937 and KG-1) on its own and in combination with thalidomide as a VEGF inhibitor. KG-1 and U937 cells exhibited increased apoptosis when curcumin and thalidomide were combined so that VEGF mRNA levels (A, B, C, and D) were reduced in KG-1 cells. Therefore, curcumin and thalidomide worked synergistically [320].

A study with selenium-L-methionine (SLM) alone or in combination with Bevacuzimab (ANV Antibody) also showed that SLM reduced AML in human cells and mice. With 400–500 nM of SLM, the combination of SLM and Bevacuzimab remarkably induced apoptosis and reduced viable cell numbers by 67–92%. Furthermore, the anti-tumor activity of SLM and bevacuzimab combined treatment was 75% more effective than that of BV alone (62% reduction) after 21 days of treatment in mice [321].

Furthermore, several monoclonal antibodies (HuMV833), small molecule VEGFR kinase inhibitors (SU5416, SU6668, SU11248, and ZD6474 in addition to soluble VEGF receptors (VEGF-Trap) and ribozymes (AngiozymeTM) have been investigated by various cancer therapy companies for their ability to inhibit VEGF or VEGFR in cancer therapy for AML [322].

The auto/paracrine effect of VEGF on AML cells brings on PI3K/AKT/endothelial NOS/NO signaling pathway which results in the proliferation and survival of AML cells [323].

In AML cell lines, Apatinib alone and in combination with HHT were studied. As a result of suppressing VEGFR2 expression and downstream signaling cascades, such as PI3K, MAPK, and STAT3, Apatinib and HHT inhibited cell proliferation, reduced the capacity for colony formation, and induced apoptosis and cell cycle arrest in AML cells [324].

VEGF autocrine activity in AML cells is divided into two types: internal and external. Both internal and external activation of VEGFR activates NF-κB, while internal pathway also activates Akt and extracellular signaling-regulated kinase (ERK). Briefly, the signaling profile of external pathway includes PI3K/AKT (cell survival), BCL-2-associated athanogene (Bag-1)/heat shock protein (HSP) (chemotherapy resistance), Ras/RAF/MEK/ERK/MSK, Ras/MKK/P38a/NF-κB (proliferation) [317]. The VEGF/VEGFR/PI3K/AKT signaling pathway continues to cause AML cell survival through several mechanisms. For example, this signaling pathway regulates BCL-2 activity while the same pathway inhibits Bax activity. In addition, positive mTOR regulation is induced by the VEGF/VEGFR/PI3K/AKT signaling pathway, which prevents Autophagy [325]. Furthermore, the auto/paracrine effect of VEGF on VEGFR-3 enhances JNK/AP-1 signaling pathway, leading to the production of COX-2, which is involved in angiogenesis [326, 327].

A number of JNK inhibitors have been investigated as treatment options for leukemia and AML, including Shikonin, Polyphyllin I (PPI), Astragalin heptacetate (AHA), Hinesol, and Parthenolide (PLT) [328].

Furthermore, endothelial cells secrete growth factors such as granulocyte colony-stimulating factor (G-CSF), granulocyte–macrophage colony-stimulating factor (GM-CSF), and Interleukin-6 (IL-6) under the influence of VEGF paracrine activity that results in the proliferation and survival of AML cells [329]. One of the reasons for survival in AML cells is the constitutive activity of STAT3 in light of the IL-6 activity [330, 331].

CXCL12 is constitutively produced by BM stromal cells and affects its receptor CXCR4 which is highly expressed on AML cells. PI3K/AKT and ERK/MAPK signaling pathways are two stimulated pathways that increase survival by activation of anti-apoptotic factors. Significantly, when the FLT3 receptor mutates, there are several signaling pathways such as STAT5, ERK/MEK and PI3K that have a synergistic effect on the CXCL12 signaling pathway and prevent apoptosis due to this mutation event [332, 333].

Studies have proven that chromosomal translocation mutation in AML cells increases Wnt signaling pathway in favor of target gene transcription [14] that leads to proliferation, survival, and differentiation of AML cells. In addition to chromosomal translocation mutation, a mutation in the FLT3 gene and overexpression of the frizzled (Fzd) receptor are involved in the overactivation of the Wnt signaling pathway [334, 335]. Interestingly, high levels of β-catenin in AML patients are associated with the FLT3 mutation [336]. Due to the binding of Wnt to its Fzd receptor, β-catenins accumulate in the cytoplasm and translocate to the nucleus, binding to TCF/LEF transcription factors and transcription target genes such as c-Myc. While FLT3-ITD signaling pathway induces the expression of a type of Fzd that is stimulated without the need for a ligand. This pathway is also responsible for the stability of β-catenin and overactive TCF [337].

A number of clinical studies in various phases have been conducted on anti-Wnt signaling inhibitors such as F2.A (anti-Frizzled receptors) and small molecules such as RXC004, CWP232291 (CBP/β-catenin antagonist), and SM04755 (WNT inhibitor) in different cancer types may lead to further understanding of AML treatment or prolongation [338, 339].

Growth factors, hormones, cytokines, and neurotrophic factors are several ligands of receptor tyrosine kinase (RTK) [340, 341]. RTK autophosphorylation, which causes subsequent reactions, results from the dimerization of two RTK monomers that occur when the ligands arrive [342]. ERK/RSK signaling pathway is activated and controls the proliferation and survival of AML cells [340]. In addition to the ERK/RSK signaling pathway, RTK potentially activates P85 which leads to sequential activation of PDK1/AKT/mTOR [204]. In addition, cell-to-cell interactions can activate RTKs such as G protein-coupled receptors (GPCRs) [343]. It seems GPCRs can participate in the RTK-dependent signaling pathways in three ways: (a) Gα and/or Gβγ subunits activate MMPs such as ROS and send them to the extracellular surface of AML cell membranes. ROSs cleavage the proligands, then the ligands are ready to bind. (b) Another possibility is that GPCR activity causes activation of P47phox. Importantly, P47phox promotes activation of ROS with the help of O2 and nicotinamide adenine dinucleotide phosphate (NADPH), subsequently, ROS inhibits the phosphorylation activity of phosphotyrosine-phosphatase (PTP), and in contrast, the phosphorylation activity of phosphotyrosine-kinase (PTK), and then PTK phosphorylates the internal domain of RTK. (c) Intracellular phosphorylation of RTK maybe be mediated by C-src cytosolic tyrosine kinase activity that originated from GPCR activation [344]. Obviously, the mutations in RTKs have an influential role in several cancers [341], especially mutations in RTK III (FLT3) that occur frequently in AML cancers [345]. Similarly, a mutation in RKT causes overactivation of the PI3K/AKT/mTOR signaling pathway [340].

Hedgehog (HH) signaling pathway is crucial in embryonic development that proliferation, survival, and differentiation of embryonic cells largely depend on this pathway, and generally in adult cells is responsible for maintenance and reconstruction [346]. However, dysregulation of HH pathway is related to several solid cancers (e.g. small-cell lung, human pancreatic carcinoma, etc.) and hematologic cancers [347], and as a result of inhibition of this pathway are very effective in apoptosis and the response to treatment [348], it seems that HH pathway is very important in drug resistance in AML [346]. In absence of ligand, HH glioma-associated oncogene (HH-GLI1) signaling pathway promotes favor of prevention of translation of target genes. To that end, when HH ligands such as Indian HH (IHH), desert HH (DHH), and sonic HH (SHH) that bind to protein patched homolog 1 (PTCH-1) are not present in the environment, PATCH-1 can bind to the seven-transmembrane protein, smoothed (SMO) and prevent its transfer to the primary cilium. This lack of transfer causes GHI-1,2,3 to be released from the primary cilium along with the suppressor of fused (SUFU) and get phosphorylated by casein kinase 1 (CK1), protein kinase A (PKA), and glycogen synthase kinase 3 β (GSK3β). Finally, this phosphorylation results in the destruction of GLI in the proteasome. The GLI 2/3 repressor (GLI2 / 3R), which results from GLI processing in the proteasome, prevents gene transcription [349–353]. In absence of the ligand, When PTCH-1 binds to its ligand, it loses the ability to bind to SMO, and hence SMO migrates to the primary cilium [354]. Likewise, phosphorylation of SMO in primary cilium leads to the separation of GLI from SUFU. Moreover, this phosphorylation inhibits PKA activity [355]. In conclusion, what enters the nucleus is the activator version of GLI (GLIA) and acts as a transcription factor. Importantly, the activity of some signaling factors can overactivate this signaling pathway. For instance, according to studies, the activity of STAT5 is related to the overexpression of GLI2 in AML cells [356]. Activation of STAT5 may occur by GLI activity. Notably, GLI not only acts as a transcription factor but also can cause STAT5 activation indirectly. However, it should also be noted that mutations in FLT3 with RAF/MEK/ERK activate STAT5 in AML cells. On the other hand, GLI also develops resistance to ribavirin and Ara-C via increasing UDP glucuronosyltransferase (UGT1A). To overcome this drug resistance administration of vismodegib has been effective in AML [354, 357]. Furthermore, in hematologic malignancies activity of BM stromal cells are in the first line of dysregulation of this signaling pathway due to increased secretion of HH ligands [356].

A new oral agent, Glasdegib, is inhibiting the Hh pathway by interfacing with smoothened proteins and inhibiting the growth of AML and human leukemia stem cells in vitro, in vivo, and in clinical trials. AML patients with C > 75 years or comorbidities preventing intensive induction chemotherapy were approved by the FDA for treatment with glasdegib in combination with LDAC [358].

## Therapeutic perspectives and future directions

Patients with AML are characterized by the generation of dysfunctional leukemic blasts, and they often suffer from life-threatening anemia and infections due to insufficient normal myelo-erythropoiesis. The physical overcrowding of BMM by accumulating leukemic cells is not the only cause of this hematopoietic failure. A variety of approaches designed to support endogenous hematopoiesis could reduce infection and anemia and thus, reduce the need for transfusion products during the management of AML patients, which usually come with several risks, including incompatibility, costs, and availability [93].

In AML, leukemic hematopoiesis is constantly adapting to environmental conditions through clonal evolution. In recent years, it seems that this both involves adaptive alterations in transcriptional control and signaling and changes in feeding and metabolizing BMM and leukemic cells. Adaptation to environmental situations drives substantial variations in the AML BMM cell dependence on lipids, carbohydrates, and amino acids. Furthermore, the processing of these metabolites occurs in the preformed metabolism circuits. These adaptations can lead to AML progression [57]. Not only metabolic checkpoints but also immune checkpoints (ICs) expressed by AML BMM and their signaling pathways can facilitate the progression, angiogenesis, metastasis, and cell proliferation in AML.

Therefore, as the BMM cells and their metabolic/immune checkpoints, as well as signaling receptors/pathways, play a vital role in the progression of AML, they can be focused on as therapeutic approaches against AML. In this line, the potential anti-AML therapeutic drugs and their targets are listed in Table 1.

**Table 1:** Potential anti-AML therapeutic approaches with targeting signaling pathways, and metabolic/immune checkpoints

Anti-cancer drug category	Drug	Target	Description	Ref
Metabolic related drugs/inhibitirs	3-bromopyruvate and benitrobenrazide	Hexokinase-2	Pyruvate analogs	[359]
	2-deoxy-D-glucose (2-DG)	Hexokinase-1	Enhancing the chemotherapeutic drug cytosine arabinoside (Ara-C) sensitivity	[360]
	Sorafenib	FLT3	High reliance on glycolysis in murine lymphoid cell line Ba/F3	[361]
	6-aminonicotinamide	G6P dehydrogenase	Inducing apoptosis in AML cell lines and primary AML blasts, but not in normal hematopoietic progenitor cells	[362]
	Oleanolic acid, Carpesium abrotanoides L, Tanshinone IIA, Dioscin, Polyphyllin VI, Euxanthone, Chrysin	GLUT1, HK2, LDHA, HIF-1α, PFK1, and PKM2	Curable effects on AML cells via affecting glucose enzymes and/or transporters	[260]
	Micheliolide (MCL)	PKM2	Selectively activates PKM2 via covalent binding at cysteine424 (C424)	[264]
	Venetoclax	BCL-2 protein–protein interaction inhibitor	More efficient in combination of hypomethylating compounds ( decitabine and azacitidine) and reduced Ara-C	[363]
	2′3'dideoxycytidine	Inhibiting the mitochondrial DNA replication	A HIV reverse transcriptase inhibitor	[364]
	A2-32–0	ClpP	Decreasing the growth rate of the transplanted OCI-AML2 cell line	[365]
	ACS-010759 and ME-344	electron transport chain	Inhibit oxidative phosphorylation (OXPHOS)	[366]
	DON (6-diazo-5-oxo-l-norleucine)	enzymes that utilize Gln as substrate	Gln antagonists	[367]
	BPTES, compound 968 and CB-839	Glutaminase	Investigated in vitro, in vivo and in different clinical trials	[368]
	1) BCT-100 2) ADI-PEG 20	Arginine production	1) Recombinant arginase 2) mycoplasma-derived arginine deiminase	[369]
	Etomoxir	CPT1	Resensitizing resistant LSCs to venetoclax with azacitidine (ven/aza) therapy	[370]
	Avocatin B	FAO	Enhancing glucose and FA absorption	[371]
Signaling factors related drugs/inhibitors	Vorolanib (CM082)	tyrosine kinase receptor	Inhibiting the proliferation of human umbilical vein endothelial cells (HUVECs) and the formation of HUVEC tubes in vitro and several xenograft models	[372]
	Curcumin	VEGF	Stronger anti-apoptosis effect in combination of thalidomide as a VEGF inhibitor in KG-1 and U937 cell lines	[373]
	Selenium-L-methionine (SLM)	VEGFR	Stronger anti-cancer effect in combination of Bevacuzimab	[374]
	HuMV833, SU5416, SU6668, SU11248, ZD6474 and PTK787/ZK222584 (Vatalanib), VEGF-Trap and AngiozymeTM	VEGF	Anti-angiogenesis agents against VEGF of VEGFR in several cancer therapy	[322]
	Apatinib	expression of VEGFR2 and Its downstream signaling cascades, such as PI3K, MAPK, and STAT3 pathways	Inhibiting cell proliferation, reducing the capacity of colony-forming, and inducing apoptosis and cell cycle arrest in AML cells in combination of HHT	[375]
	Shikonin, Polyphyllin I (PPI), Astragalin heptacetate (AHA), Hinesol and Parthenolide (PLT)	JNK	-	[328]
	RXC004 and CWP232291	CBP/β-catenin	Act as CBP/β-catenin antagonist	[376]
	SM04755	WNT	Act as anti-WNT signaling agent	[377]
	Glasdegib	Hh pathway	Interacting with smoothened protein and inhibiting the growth of AML cell lines and human leukemia stem cells	[378]
Immune checkpoint related drugs/inhibitirs	Cytosine arabinoside (cytarabine)	CD80 and CD86	Inducing the expression of CD80 and CD86 and reducing the expression of PD-1 on leukemic cells, making them more susceptible to cytotoxic T-lymphocyte-mediated killing	[379]
	Ipilimumab	CTLA-4	Anti-CTLA-4 monoclonal antibody	[380]
	Pembrolizumab, nivolumab, and cemiplimab	PD-1	Anti-PD-1 antibodies	[381]
	Atezolizumab, avelumab, and durvalumab	PD-L1	Anti-PD-L1 antibodies	[382]
	TSR-022 and MBG453	TIM-3	Anti-TIM-3 monoclonal antibodies	NCT02817633 NCT02608268
	Daratumumab and Isatuximab	CD38	Anti-CD38 antibodies	[158]
	Poliovirus-Rhinovirus Chimera (PVSRIPO)	CD155	An oncolytic viral therapy	[383]
	OMP-313M32, BMS-986207, MTIG7192A and MTIG7192A	TIGIT	Their alone or combination effects with nivolumab and atezolizumab have been clinically investigated	[384]
	TTI-CD200	CD200	an anti-CD200 antibody investigated on leukemia-propagating cells (LPCs) and mice models	[233]

## Conclusions and future perspectives

AML is a very heterogeneous and complex group of diseases, and despite recent advances in therapeutic strategies, the standard treatment still has a high rate of resistance. Therefore, it is critical to find innovative approaches and treatments for patients with AML. In this review, we summarized metabolic and immune checkpoint characteristics of AML BMM, as a major cause of AML progression, angiogenesis, metastasis, and cell proliferation. In this regard, metabolic and immune checkpoints of AML BMM cells and their downstream stimulated signaling pathways may be considered as prognostic and therapeutic targets for AML. Consequently, acquiring knowledge regarding the functions and mechanisms of action of BMM cells, including their involvement in immune and metabolic checkpoints, as well as their capacity for the production of components and stimulation of signaling pathways, could provide a promising outlook for the development of novel therapeutic interventions aimed at targeting multiple factors in acute myeloid leukemia (AML) that are influenced by BMM cells. Henceforth, future research should undertake a comprehensive clarification of the importance of these metabolic and immune checkpoints of AML BMM cells on leukemic cells. Furthermore, these cells and checkpoints should be deemed as feasible multi-targeted therapies for AML in conjunction with other conventional treatments for AML.

## Data Availability

Not applicable.

## Abbreviations

AML: Acute myeloid leukemia
BM: Bone marrow
BMM: Bone marrow microenvironment
TME: Tumor microenvironment
ICs: Immune checkpoints
CML: Chronic myeloid leukemia
MM: Multiple myeloma
CLL: Chronic lymphocytic leukemia
CRCs: Colorectal cancers
FLT3-ITD: Fms-like tyrosine kinase 3-internal tandem duplication
KIRs: Killer cell immunoglobulin-like receptors
NCRs: Natural cytotoxicity-activating receptors
PD-L1: Programmed death-ligand 1
PD-1: Programmed cell death protein 1
MDS: Myelodysplastic syndrome
EVs: Extracellular vehicles
BMAT: BM adipose tissue
Gal-9: Galectin-9
RTK: Receptor tyrosine kinases
HSPCs: Hematopoietic stem/ progenitor cells
TAM: Tumor associated macrophage
NK: Natural killer
DCs: Dendritic cells
mDCs: Myeloid dendritic cells
pDCs: Plasmacytoid dendritic cells
MDSCs: Myeloid-derived suppressor cells
LICs: Leukemia-initiating cells
CAFs: Cancer-associated fibroblasts
Tregs: Regulatory T cells
CAR: Chimeric antigen receptor
ECM: Extracellular matrix
MMPs: Matrix metalloproteinases
TGF-β: Transforming growth factor-β
MIP-l α: Macrophage inflammatory protein-1α
Ang: Angiopoietin
SCF: Stem cell factor
VEGF: Vascular endothelial growth factor
PDGF: Platelet-derived growth factor
SDF: Stromal cell derived factor
SIRPα: Signal Regulatory Protein Alpha
PB: Peripheral blood
PTCH-1: Protein patched homolog 1
TNF-α: Tumour Necrosis Factor alpha
NF-κB: Nuclear factor kappa B
STAT: Signal transducer and activator of transcription
HIF-1α: Hypoxia-inducible factor-alpha
mTOR: Mammalian target of rapamycin
mTORC1: Mammalian target of rapamycin complex 1
ERK: Extracellular signaling-regulated kinase
Arg: Arginine
IDO: Indoleamine 2,3-dioxygenase
HA: Hyaluronic acid
GLUD: Glutamate dehydrogenase
BCAA: Branched-chain amino acid
FAO: Fatty acid β-oxidation
ROS: Reactive oxygen species
NADPH: Nicotinamide adenine dinucleotide phosphate
CK1: Casein kinase 1
SUFU: Suppressor of fused
GrB: Granzyme B
GPI: Glycosylphosphatidylinositol
GLUTs: Glucose transporters
MCTs: Monocarboxylate transporters
α-KG: α-Ketoglutarate
PPP: Pentose phosphate pathway
